# Investigating the mechanisms of indocyanine green tumour uptake in sarcoma cell lines and *ex vivo* human tissue

**DOI:** 10.1002/path.6473

**Published:** 2025-09-10

**Authors:** Corey David Chan, Marcus J Brookes, Toni A Pringle, Rahul Pal, Riya Tanwani, Alastair D Burt, James C Knight, Anand TN Kumar, Kenneth S Rankin

**Affiliations:** ^1^ The North of England Bone and Soft Tissue Tumour Service Newcastle upon Tyne Hospitals NHS Foundation Trust Newcastle upon Tyne UK; ^2^ Newcastle University Centre for Cancer Translational and Clinical Research Institute, Newcastle University Newcastle upon Tyne UK; ^3^ School of Natural and Environmental Sciences Newcastle University Newcastle upon Tyne UK; ^4^ Department of Radiology Massachusetts General Hospital, Harvard Medical School Boston MA USA; ^5^ Department of Otolaryngology‐Head and Neck Surgery Massachusetts Eye and Ear, Harvard Medical School Boston MA USA; ^6^ Newcastle University Medical School Newcastle University Newcastle upon Tyne UK; ^7^ NovoPath, Department of Cellular Pathology Newcastle upon Tyne Hospitals NHS Foundation Trust Newcastle upon Tyne UK

**Keywords:** indocyanine green, sarcoma, osteosarcoma, fluorescence‐guided surgery, fluorescence imaging, near‐infrared, surgical oncology, fluorescence microscopy, histology

## Abstract

Indocyanine green (ICG) is a well‐established near‐infrared dye which has been used clinically for several decades. Recently, it has been utilised for fluorescence‐guided surgery in a range of solid cancer types, including sarcoma, with the aim of reducing the positive margin rate. The increased uptake and retention of ICG within tumours, compared with normal tissue, gives surgeons a visual reference to aid resection when viewed through a near‐infrared camera. However, the mechanisms of this process are poorly understood. We performed *in vitro* ICG cellular uptake studies across a panel of sarcoma cell lines exhibiting varying proliferation rates and phenotypes. The effects of ICG concentration, incubation time, inhibition of clathrin‐mediated endocytosis, and cell line proliferation rate on the cellular uptake of ICG were investigated using fluorescence microscopy and flow cytometry. Subcellular localisation of intracellular ICG was assessed *via* colocalization with a lysosomal marker. The spatial distribution of ICG in patient tumour tissue following fluorescence‐guided surgery was assessed by high‐resolution tissue imaging and quantified using fluorescence lifetime imaging. *In vitro* results showed that the cell line proliferation rate correlated significantly with ICG uptake (Spearman's rank correlation coefficient = 1.00, *p* < 0.001), and maximum ICG uptake was observed after 24 h incubation. ICG cellular uptake was significantly reduced by inhibition of clathrin‐mediated endocytosis (*p* = 0.0004), and intracellular ICG significantly colocalized with a lysosomal marker within 30 min (Pearson's *r* = 0.8). On histological analysis of tumour tissue from three different sarcoma subtypes, ICG was observed within sarcoma cells as well as accumulating in paucicellular areas of haemorrhage and necrosis within the tumour microenvironment. Through quantification of fluorescence lifetime imaging of ICG, we were able to differentiate sarcoma cells from haemorrhage and necrosis within tumour tissue. Combining *in vitro* data with analysis of patient tissue, we propose that the uptake and accumulation of ICG in sarcomas is driven by a synergistic mechanism involving the enhanced permeability and retention effect combined with active tumour cell endocytosis of the dye. © 2025 The Author(s). *The Journal of Pathology* published by John Wiley & Sons Ltd on behalf of The Pathological Society of Great Britain and Ireland.

## Introduction

Indocyanine green (ICG) is a near‐infrared (NIR) fluorescent tricarbocyanine dye, first adopted clinically for retinal vein imaging, cardiac output studies, and the assessment of hepatic function [1, 2, 3]. It has a well‐established safety profile and has been approved for clinical use by both the US Food and Drug Administration and the European Medicines Agency [4]. ICG has previously been used for lymph node mapping and angiography, and more recently is being trialled for fluorescence‐guided surgery (FGS) [5, 6, 7]. FGS techniques following administration of ICG include evaluation of skin flap viability and congestion [8, 9, 10], visualisation of healthy versus ischaemic bowel in colorectal surgery [11], and the identification of sentinel lymph nodes in breast [12, 13], gastrointestinal [14, 15, 16], and gynaecological malignancies [17]. Recently, ICG is being used to identify malignant tumours and has been shown to accumulate in multiple solid cancer types resulting in fluorescence of the tumour when viewed with an NIR camera [5]. This was first described in hepatocellular carcinomas and associated metastatic lesions [18, 19], and has since been described for ovarian cancer [20], head and neck cancer [21], lung cancer [22], breast cancer [23], and bone and soft‐tissue sarcomas arising in the extremities and pelvis, including chondrosarcomas, myxofibrosarcomas, leiomyosarcomas, synovial sarcomas, and osteosarcomas [24]. Our recent case series of 115 patients evaluating the positive margin rates following sarcoma resection using FGS with ICG (*n* = 39), versus standard resection alone (*n* = 76), revealed a significantly lower positive margin rate in the FGS group [25]. The use of ICG for sarcoma is therefore of high interest, with the aim of improving surgical margins and reducing local recurrence rates; however, its recent clinical adoption in sarcoma surgery has prompted the need for further basic science research to inform optimal dosing, administration, and imaging of ICG within these tumours.

The optimal timing of ICG administration for tumour surgery is unclear, with reported protocols varying widely from immediately prior to surgery [23] to 28 days preoperatively [26]. Other studies suggest that an optimal tumour‐to‐background signal is achieved when ICG is administered 24 h prior to the procedure [27, 28], which is also supported for the paediatric sarcoma population [29]. The optimal dose of ICG is also yet to be identified; a recent paediatric trial assessing its use for tumour surgery, including sarcomas, has implemented weight‐based dosing with the aim of standardising the bioavailability of ICG at the tumour site [30]. The uncertainty surrounding both the dose and timing of ICG for tumour surgery is due to limited clinical studies, and a lack of understanding of its mechanism of action [31].

ICG has well‐established absorption and emission properties within the NIR spectral range of 780–830 nm; however, these properties vary depending on its concentration and solvent [32, 33, 34]. NIR wavelengths have excellent tissue penetration [3], which makes ICG well suited to sarcoma surgery since the tumours are typically deep and covered by multiple tissue layers, often including a pseudocapsule that contains the bulk of the tumour. ICG readily binds to lipoproteins in circulation and has a plasma half‐life of 2–4 min [35]. It is rapidly and exclusively excreted *via* the biliary system and does not undergo enterohepatic circulation [36, 37, 38]. Despite the well‐established pharmacokinetic and structural properties of ICG, the mechanisms by which the dye accumulates within and is retained by tumours are not well understood, as unlike targeted dyes, ICG does not exhibit a specific cancer‐cell targeting moiety [39]. From preclinical models, the accumulation of ICG within tumours is thought to be primarily attributable to the enhanced permeability and retention (EPR) effect [40], which describes the extravasation and retention of macromolecules in tumours due to irregular neovascularisation, aberrant blood vessel endothelia, and impaired lymphatic drainage [41]. Maeda *et al* postulated that, as ICG readily binds to albumin and globulin *in vivo*, it selectively extravasates only through the leaky tumour vasculature [40]. A study by Onda *et al* challenged this theory with the finding that ICG rapidly extravasates in all tissues in a colorectal cancer mouse model, with no tumour‐specific delivery demonstrated [42]. Instead, that study suggested that preferential tumour cell uptake of ICG was due to the high endocytic rate of cancer cells [42], and may be related to the affinity of ICG to phospholipids and its inherent ability to bind to cell membranes prior to endocytosis [43]. The work suggested that, overall, the primary mechanism of tumour fluorescence involved increased tumour cell uptake and intracellular retention of ICG, as opposed to the EPR effect. In contrast, recent work by Sardar *et al* found ICG in higher concentrations in the acellular, necrotic areas in a mouse model of synovial sarcoma [44]. With these conflicting findings, it is apparent that the mechanisms for the increased tumour uptake and accumulation of ICG, compared to non‐tumour tissue, are still unclear. There have been very few previous studies looking into the spatial distribution of ICG in tissue obtained from sarcoma patients who have undergone FGS with ICG [45].

From a clinical perspective, innovative widefield NIR imaging technology has allowed intraoperative assessment of tumour margins with fluorescent agents [46]. A recent international consensus from experts across five continents concluded that FGS with ICG is both effective and safe, but further research is required to optimise its use [31]. Despite recent advancements in novel targeted NIR dye conjugates for FGS in some cancer types [47, 48], the fact that ICG is safe, rapidly cleared, readily available, and easy to administer makes it an appealing dye for current FGS practices. Two UK randomised controlled trials, SarcoSIGHT [6] and GLO‐Surgery [7], are currently investigating whether ICG is beneficial for patient outcomes. An improved understanding of the mechanisms of ICG uptake will inform clinical use of the dye, helping to optimise dose, route, and timing of administration. Consequently, our aim was to further understand the mechanisms of ICG tumour uptake in sarcoma by assessing cellular uptake, retention, and subcellular localisation *in vitro*, alongside high‐resolution NIR tissue imaging from sarcoma patient samples.

## Materials and methods

### Ethics approval and consent to participate

Appropriate informed consent for the use of tumour specimen slides and paraffin‐embedded blocks was obtained from all patients. This study was approved by the Newcastle and North Tyneside 1 Research Ethics Committee (REC Reference Number: 17/NE/0361). Informed consent for the use of patient tissue was obtained from all subjects involved in the study.

### Cell culture

Four human sarcoma cell lines HT‐1080 (dedifferentiated chondrosarcoma), U2OS (osteosarcoma), MG‐63 (osteosarcoma), and SaOS‐2 (osteosarcoma) were used in this study, along with the low proliferating human carcinoma cell line MCF‐7 (breast carcinoma) as a comparative control. All cells were cultured in RPMI 1640 medium (Sigma‐Aldrich, Dorset, UK) supplemented with 10% foetal bovine serum (Gibco, London, UK), 100 U penicillin/ml, and 0.1 mg streptomycin/ml (Gibco), incubated at 37 °C, 5% CO_2_ humidified atmosphere. For Pitstop 2 experiments, serum‐free DMEM/F‐12 with 15 mm HEPES (Gibco), supplemented with 100 U penicillin/ml and 0.1 mg streptomycin/ml (Gibco) were used. Further details are provided in supplementary material, Table S1.

### Fluorescent agents

Clinical‐grade ICG 25 mg lyophilised powder (Verdye, Diagnostic Green, Aschheim‐Dornach, Germany) was dissolved in cell culture medium (10 ml) and sterile filtered using a 0.22 μm Minisart Plus Syringe Filter (Sartorius, Surrey, UK). The ICG solution was diluted to achieve a 100‐μm stock solution, which was stored in the dark at 4 °C and used for a maximum of 5 days. Cell nuclei were stained with mounting medium with DAPI Aqueous Fluoroshield (Abcam, Cambridge, UK). LysoTracker Deep Red (L12492, 1 mm, Invitrogen, Paisley, UK) was diluted in culture medium to achieve a working concentration of 50 nm.

### Cell preparation in chamber slides

Between 2 × 10^4^ and 4 × 10^4^ cells/well were seeded in Nunc® Lab‐Tek® II CC2 chamber slides (Thermo Fisher Scientific, Leicestershire, UK) and left to establish for 48 h at 37 °C, based on the proliferation rate of each cell line to ensure a consistent number of cells at the time of experimentation (supplementary material, Table S2). At 48 h, the media was aspirated and cells were incubated in ICG in media (400 μl) for either 15 or 30 min. ICG‐containing media was removed, and cells washed with media ×2, before being fixed with 400 μl 4% paraformaldehyde (PFA) solution (Sigma‐Aldrich) for 10 min at room temperature (RT). PFA was removed, cells washed ×3 in PBS, the chamber system was removed, and the cells were stained with DAPI mounting medium (Abcam) before adding a glass coverslip.

### Fluorescence microscopy

Slides were imaged immediately after preparation using the Zeiss AxioImager widefield microscopy system (Carl Zeiss Microscopy Ltd, Cambridge, UK), at 20× and 40× magnification. Excitation and emission filters: DAPI (Cy2), 335–385 nm/420–470 nm; ICG (Cy7) 670–745 nm/768–850 nm; LysoTracker (Thermo Fisher Alexa Fluor 647) 625–655 nm/665–715 nm. Further details are provided in the Supplementary materials and methods.

### Image quantification

The 40× magnification images were quantified by calculating the mean fluorescence intensity per cell. The .CZI files were opened in ZEN Blue Edition software v3.3 (Zeiss Group Headquarters, Oberkochen, Baden‐Württemberg, Germany), with the Cy2, Cy7, and DIC channels overlayed. The polygonal tool was used to draw around each cell guided by the DIC overlay, and the arithmetic mean of Cy7 signal was generated by the ZEN software (supplementary material, Figure S1). FLIM quantification of extracellular versus intracellular ICG from patient tissue was performed using Fiji ImageJ (v2.3.0/1.53, https://imagej.net/software/fiji/downloads, date last accessed 1 May 2025) using four regions of interest (ROIs) from FLIM microscopy images (supplementary material, Figure S2).

### Flow cytometry

Cells were seeded in a 6‐well plate at a density between 2.5 × 10^5^ and 5 × 10^5^ cells/well and left to establish for 48 h at 37 °C, achieving cell confluency of 80%–90%. Cells were incubated with ICG (25 μm) in normal media (400 μl) for the desired time period (15 min, 30 min, 24 h, 48 h) at 37 °C. ICG was removed, washed x3 with PBS, trypsinised, and neutralised in media. The cell pellets were suspended in 500 μl ice‐cold flow buffer (0.5% bovine serum albumin, 2 mm EDTA (Sigma‐Aldrich) in phosphate‐buffered saline [PBS]), for analysis on FACSCanto II (BD Biosciences, Berkshire, UK) with a bandpass filter 635 nm, 780/60. For the 24 h retention experiment ICG was removed after 30 min, and cells incubated for an additional 24 h at 37 °C in fresh media before processing. Flow cytometry data for ICG cellular uptake studies was analysed using FlowJo v10.8 Software for MacOS (Becton, Dickinson and Company, Franklin Lakes, NJ, USA), (supplementary material, Figures S3–S5).

### Cellular proliferation rate

Cellular proliferation rates were assessed using Cell Counting Kit‐8 (Dojindo Laboratories, Mashikimachi, Japan) following the manufacturer's protocol [49]. All cell lines were seeded at a density of 1.5 × 10^4^ cells per well in a 96‐well plate in triplicate alongside a media‐only control and incubated at 37 °C for 48 h. CCK‐8 solution (10 μl) was added to each well and incubated at 37 °C for 2 h, then absorbance was measured at 450 nm using an Omega FLUOstar (BMG LABTECH, Swindon, UK) microplate reader.

### Inhibition of clathrin‐mediated endocytosis

For microscopy, HT‐1080 cells were prepared in Nunc® Lab‐Tek® II CC2 chamber slides (Thermo Scientific) at a density of 2 × 10^4^ cells/well and left to establish for 48 h. Cells were incubated in serum‐free media for 10 min prior to the experiment. Cells were incubated with 5–30 μm of Pitstop 2 (Abcam; ab120687) or 30 μm Pitstop 2 negative control (Abcam; ab120688) diluted in serum‐free media containing 0.1% DMSO for 15 min at 37 °C. After 15 min, ICG in serum‐free media (50 μm) was added to preexisting media to achieve a final ICG concentration of 25 μm/well and incubated for 30 min at 37 °C, in the continued presence of the inhibitor. For flow cytometry, cells were seeded in a 6‐well plate at a density of 2.5 × 10^5^ cells/well, treated with Pitstop 2, as above, and incubated with ICG (10 μm or 25 μm) for 30 min.

### Lysosomal staining and colocalisation with ICG

HT‐1080 cells were prepared in Nunc® Lab‐Tek® II chamber slides at a density of 2 × 10^4^ cells/well. After 48 h, media was removed and 50 nm of LysoTracker Deep Red (L12492, Invitrogen) in 400 μl of media was added and incubated at 37 °C for 1 h. Lysotracker was removed, and the cells washed with 400 μl media. Cells were incubated with ICG (10 μm) for 30 min and prepared for imaging as stated previously. CZI image files were analysed using Fiji ImageJ (v2.3.0/1.53), (supplementary material, Figure S6).

### Patient tumour sections

Tumour samples from three sarcoma patients were analysed: a high‐grade osteosarcoma, a grade 3 leiomyosarcoma, and a grade 2 chondrosarcoma. Patients received 75 mg ICG administered intravenously either 16 h preoperatively (osteosarcoma and chondrosarcoma) or at induction of anaesthesia (leiomyosarcoma). Following resection, the specimen was visualised with a Stryker SPY‐PHI infrared camera (Stryker Corp, Kalamazoo, MI, USA). Tumour specimens were processed by histopathology and 3‐μm sections cut from FFPE tissue blocks using a Leica RM2245 microtome (Leica Biosystems, Milton Keynes, UK) and mounted on SuperFrost Plus adhesive slides (Thermo Fisher Scientific). Hematoxlyin and eosin (H&E) staining was performed on a Dako CoverStainer automated platform. For IHC staining, MT1‐MMP antibody (MAB3328/*MMP‐14* clone LEM‐2/15.8, Merck Life Science UK Limited, Dorset, UK) was diluted 1:2,500, and CD45 antibody (M0701/clone 2B11 + PD7/26, Agilent Technologies LDA UK Limited, Cheshire, UK) was diluted 1:250. Antigen retrieval was performed using Discovery CC1 06414575001/pH 8.5 (Roche Diagnostics, West Sussex, UK) applied at 100 °C for 32 min. Further details are provided in the Supplementary materials and methods, and primary antibody and reagent information are available in supplementary material, Table S3.

### High‐resolution tissue imaging and biomolecular imaging

Patient ICG specimen sections were imaged using a VS200 slide scanner (Olympus, Hamburg, Germany), with single‐band excitation and emission filters: DAPI; 352–404 nm/416–452 nm, and ICG; 721–749/767–849 nm. Image files were analysed and processed using Olympus OlyVIA software (v3.4.1). Specimen slides were imaged macroscopically using the Typhoon NIR Biomolecular Imager (Cytiva Bioscience, Buckinghamshire, UK). Slides were scanned at a resolution of 10 μm using the Cy7 laser for ICG, and the Cy2 laser for DAPI. Image files were processed and analysed using Fiji ImageJ (v2.3.0/1.53). Fluorescence lifetime imaging (FLIM) was performed, processed, and analysed as per previously published methods [50].

## Statistical analyses

Verification of homogeneity of the variances was performed using Bartlett's test on GraphPad Prism v10.4.1 (532) for MacOS (GraphPad Software, Boston, MA, USA). Comparisons between two sample groups were analysed using Student's *t*‐test, following a Shapiro–Wilk test for normality. Analysis of correlations between continuous variables was performed using the non‐parametric Spearman's Rho function (2‐tailed) on SPSS (IBM SPSS Statistics V.27 for MacOS, Chicago, IL, USA). Colocalization analysis was performed using the Coloc 2 plugin in Fiji ImageJ (v2.3.0/1.53) with individual image thresholds automatically defined by Costes regression.

## Results

### ICG uptake in sarcoma cells is dependent on concentration and incubation time

A panel of three cancer cell lines were chosen as representative of high (HT‐1080), medium (U2OS) and low (MCF‐7) proliferative potential, for concentration and incubation studies.

**Figure 1:**
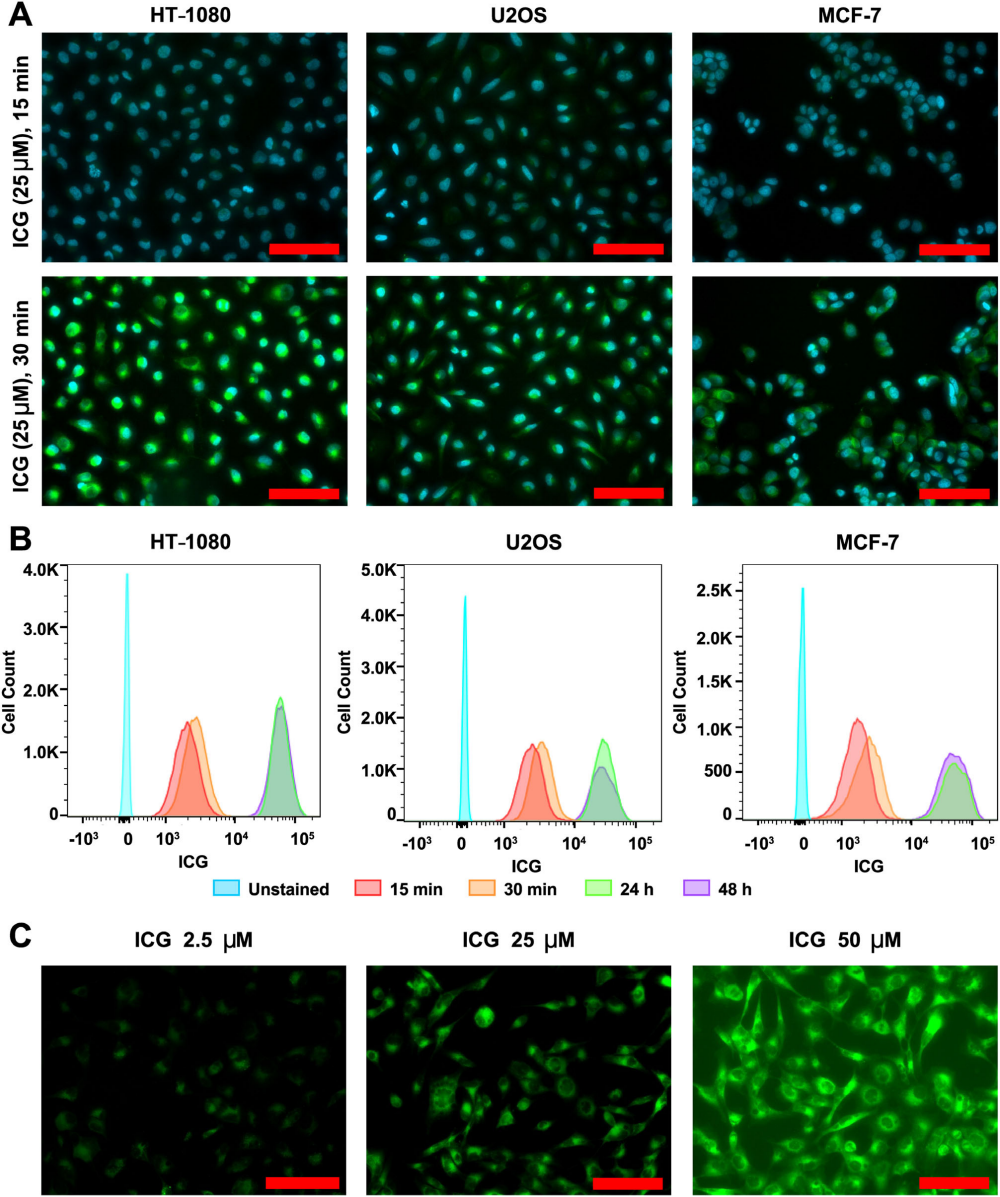
*In vitro* uptake of ICG in cancer cell lines at differing ICG concentrations and incubation times. (A) Fluorescence microscopy images of HT‐1080, U2OS, and MCF‐7 cells after incubation with 25 μm ICG (green) counterstained with DAPI nuclear stain (blue) for 15 min versus 30 min (20× magnification). (B) Flow cytometry data demonstrating ICG cellular uptake for HT‐1080, U2OS, and MCF‐7 cells incubated with 25 μm ICG for increasing time periods up to 48 h. (C) Fluorescence microscopy of HT‐1080 cells incubated with increasing ICG concentrations (2.5–50 μm); for 30 min (20× magnification). Red scale bars, 100 μm. ICG, indocyanine green.

Increasing the incubation time from 15 to 30 min led to an increase in the cellular ICG signal on fluorescence microscopy in two sarcoma cell lines of 9.2% and 6.4% for HT‐1080 and U2OS, respectively, and 5.4% for the MCF‐7 breast cancer line, when quantified by analysis of ROIs (Figure 1A). Serial timepoint incubation studies using flow cytometry demonstrated that across all three cell lines, ICG cellular uptake increased with the incubation time up to a maximum at 24 h, with no further increase seen at 48 h (Figure 1B). With increasing ICG concentration, the amount of intracellular signal after 30 min also increased; however, at 50 μm, notable background signal and oversaturation was observed on fluorescence microscopy with HT‐1080 cells (Figure 1C).

### Cellular ICG uptake correlates with cell line proliferation rate

**Figure 2:**
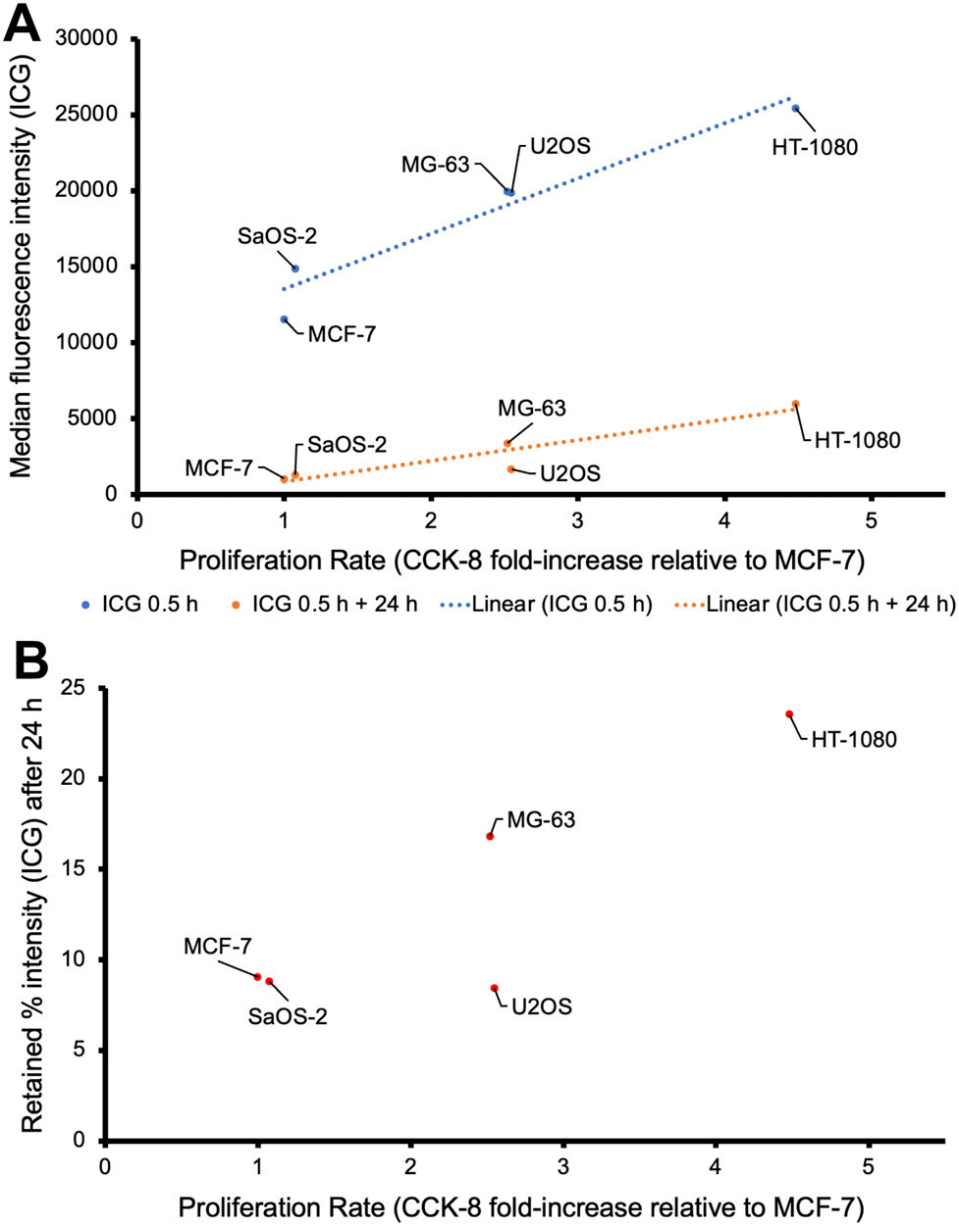
Correlation between cellular ICG uptake and cell proliferation rate. (A) Scatterplot showing the correlation between cellular ICG uptake and cell line proliferation rate, and the correlation between ICG retention after 24 h and cell line proliferation rate. Cells were incubated in 25 μm ICG for 0.5 h (blue) or 0.5 h + 24 h without ICG (orange) and analysed by flow cytometry (laser 635,780/60 nm). The cell line proliferation rate was calculated by CCK‐8. Data points are represented as mean ± SEM, with linear trend line (dotted blue). Non‐parametric Spearman's rho functions: Rs = 1.000, *p* < 0.001 for 0.5 h, and Rs = 0.9, *p* = 0.0374 for 0.5 h + 24 h. (B) Scatterplot showing the percentage of retained ICG intensity after 24 h across each cell line against proliferation rate, Rs = 0.3, *p* = 0.624. MFI, median fluorescence intensity; CCK‐8, cell counting kit‐8; SEM, standard error of the mean; ICG, indocyanine green; Rs, Spearman's rank correlation coefficient.

ICG cellular uptake varied across a panel of cancer cell lines when incubated with 25 μm ICG for 30 min, on both fluorescence microscopy and flow cytometry. The median fluorescence intensity (MFI) on flow cytometry was strongly positively correlated with the proliferation rate (CCK‐8) (Figure 2A); Spearman's rank correlation coefficient = 1.000, *p* < 0.001. The HT‐1080 cell line is traditionally reported as a ‘bone fibrosarcoma’; however, since it is now known to harbour an *IDH1* mutation it is more representative of an aggressive dedifferentiated chondrosarcoma [51]. These cells proliferate rapidly and exhibited the highest MFI after 30 min, indicating the greatest level of ICG uptake. The least proliferative sarcoma cell line, SaOS‐2, showed the lowest MFI after 30 min, alongside the MCF‐7 breast cancer cell line, which is widely considered to have low aggressiveness and low metastatic potential, and was used in this study as a relative control [52].

### Residual intracellular ICG after 24 h varies between sarcoma cell lines

Given the differences in ICG cellular uptake between sarcoma cell lines, we investigated whether the proliferation rate correlated with the retention of ICG, determined by residual fluorescence after 24 h. After 30 min incubation with 25 μm ICG, followed by 24 h in ICG‐free media, the residual ICG signal on flow cytometry followed a strong positive correlation with the cellular proliferation rate (Figure 2A); Spearman's rho = 0.9, *p* = 0.037. However, the retained intensity percentage after 24 h differed between cell lines (Figure 2B), and no longer correlated with the proliferation rate; Spearman's rho = 0.3, *p* = 0.624. The most proliferative sarcoma cell line (HT‐1080) had the highest residual fluorescence after 24 h (23.5%), whilst the lowest residual fluorescence was observed in the U2OS cell line (8.39%). The slowly proliferating SaOS‐2 and MCF‐7 cell lines demonstrated the lowest fluorescence intensity after 30 min incubation and had a low residual fluorescence of ICG after 24 h.

### Inhibition of clathrin‐mediated endocytosis reduces ICG cellular uptake

**Figure 3:**
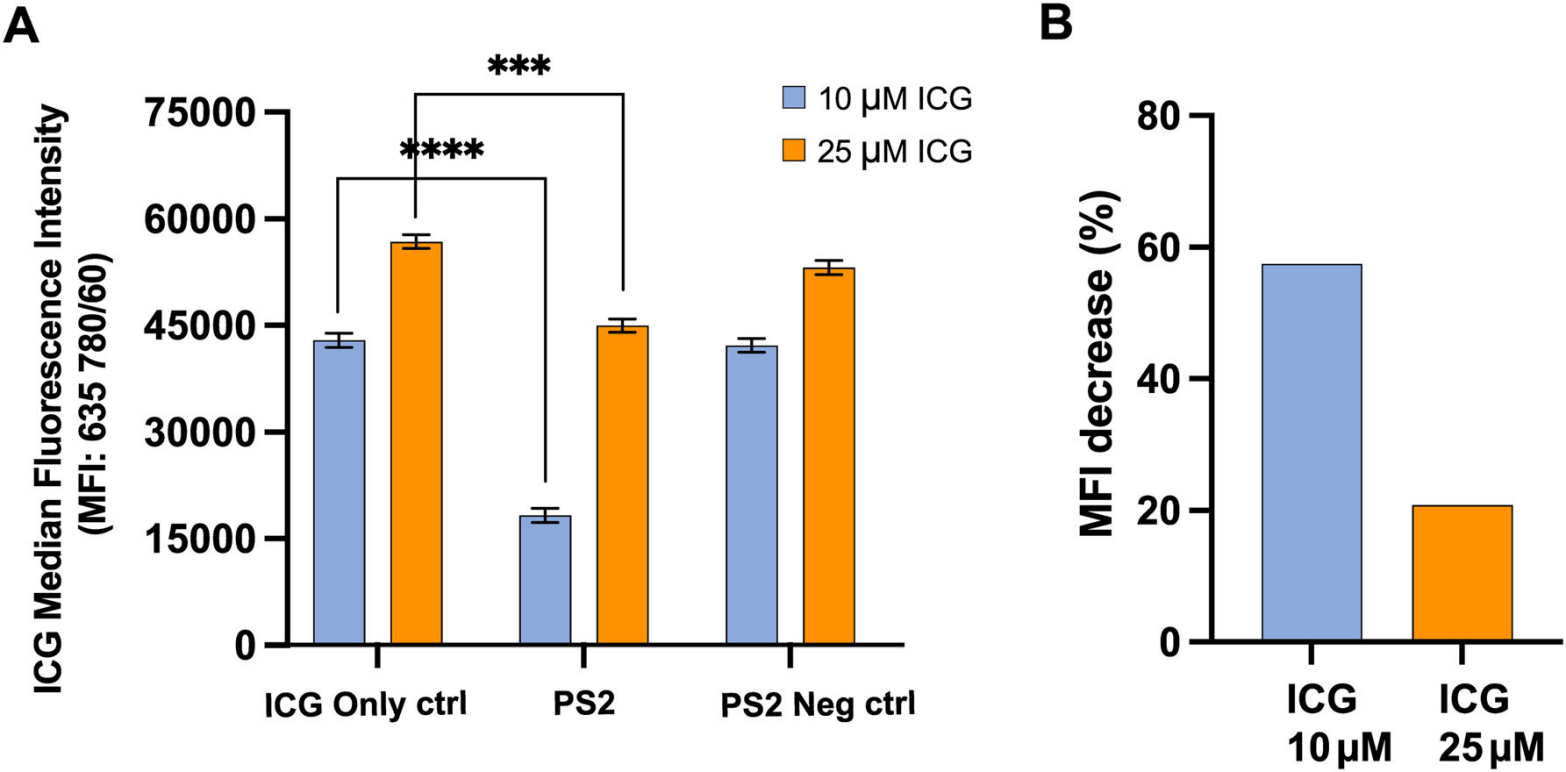
The effect of PS2 on the cellular uptake of ICG in the HT‐1080 cell line. (A) Analysed data from flow cytometry showing MFI after treatment with 30 μm PS2 inhibitor compound or 30 μm PS2 negative control compound, followed by 30 min incubation with 10 μm ICG (blue bars) or 25 μm ICG (orange bars), compared with an untreated ICG‐only control, bars represent 95% confidence intervals (CI). (B) Percentage reduction in MFI observed in HT‐1080 cells after treatment with 30 μm PS2 at differing ICG concentrations for 30 min. MFI, median fluorescence intensity; PS2, Pitstop 2; ICG, indocyanine green. Statistical analysis performed using paired *t*‐test ***p < 0.001, ****p < 0.0001.

**Figure 4:**
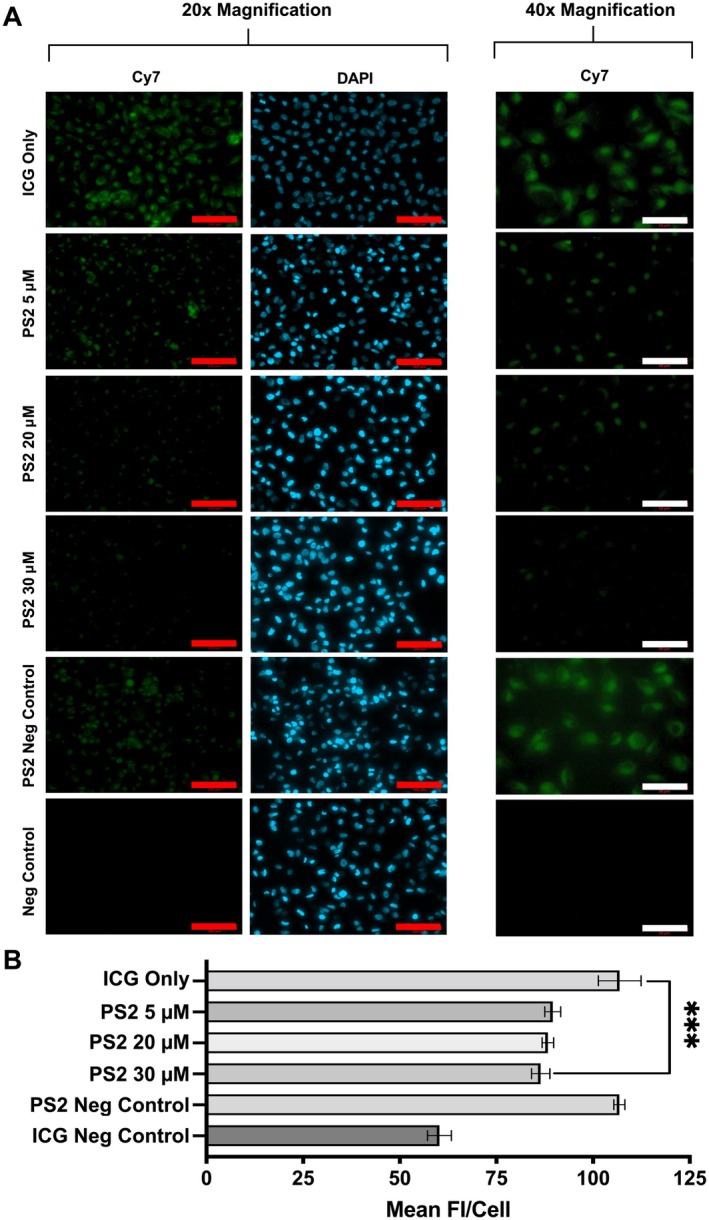
The effect of increasing concentrations of PS2 on ICG cellular uptake. (A) HT‐1080 cells were incubated with either PS2 compound (5, 20, 30 μm), PS2 negative control compound (30 μm), or normal media for 15 min, followed by incubation with 25 μm ICG for 30 min in the continued presence of the inhibitor. PS2 negative control is of the same chemical class and has a highly related structure to PS2, but does not block receptor‐mediated endocytosis at concentrations up to 300 μm. Fluorescence images at magnification 20× (left) and 40× (right) show intracellular ICG (green) at differing PS2 concentrations, with cell nuclei stained with DAPI (blue). Red scale bars, 100 μm; white scale bars, 50 μm. (B) Quantification of 40× images by calculation of mean fluorescence intensity per cell. Statistical analysis performed using paired *t*‐test; ***p < 0.001. Values are means with 95% CI.Mean FI, mean fluorescence intensity; PS2, Pitstop 2; CI, confidence intervals.

When HT‐1080 cells were preincubated with PS2, a selective inhibitor of CME, subsequent cellular uptake of ICG was significantly reduced at 30 min compared to the ICG‐only control (ICG 10 μm, *p* < 0.0001; ICG 25 μm, *p* = 0.0004) (Figure 3A); this timepoint was chosen, as a high intracellular presence of ICG was observed at 30 min in the absence of inhibition. The HT‐1080 cell line was selected, as it demonstrated the highest level of ICG uptake (Figure 2A). The effect of PS2 on ICG uptake was quantified using both flow cytometry (Figure 3) and fluorescence microscopy (Figure 4). On flow cytometry analysis, the level of PS2 inhibition was influenced by the concentration of ICG (Figure 3A,B), with a greater reduction in MFI observed when cells were incubated with lower concentrations of ICG (10 μm), compared with higher ICG concentrations (25 μm) (Figure 3B). On analysis of fluorescence microscopy images, the mean FI value per cell decreased with increasing PS2 concentration, and there was a significant difference between the PS2‐treated cells (30 μm) compared with the ICG‐only control (30 μm) (*p* < 0.001) (Figure 4B). There was no statistically significant difference between the varying concentrations of the inhibitor on quantitative image analysis (Figure 4A,B). Although PS2 significantly reduced the uptake of ICG compared with the PS2 negative control, it did not completely block ICG uptake to the level of the ICG negative control (Figure 4B).

### Intracellular ICG colocalizes with lysosomes

**Figure 5:**
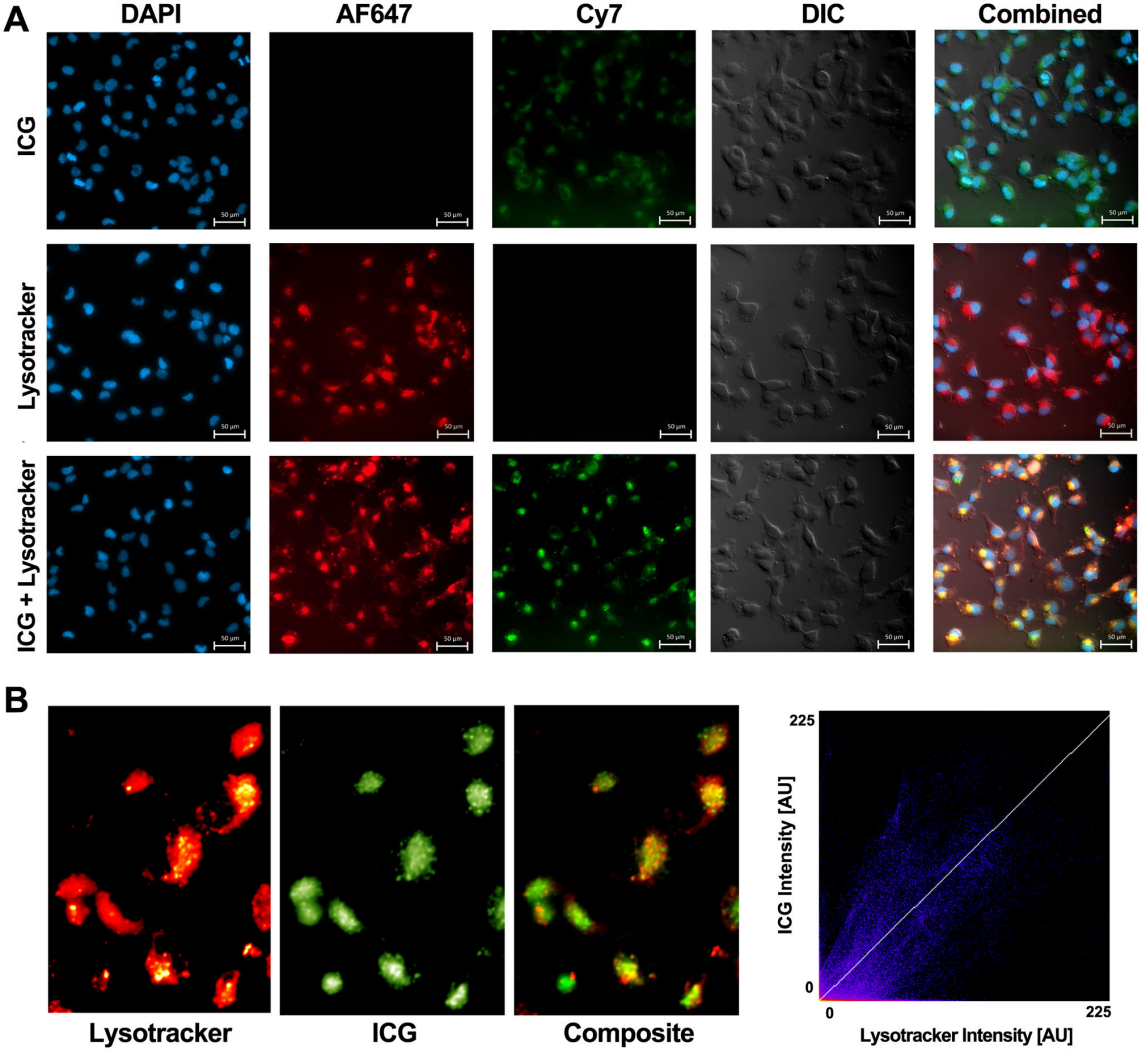
Fluorescence microscopy images of ICG and Lysotracker colocalization. (A) HT‐1080 cells incubated with 50 nm lysotracker (red) for 1 h followed by 10 μm ICG (green) for 30 min. Cells were stained with a DAPI nuclear stain (blue). Split channel images at 40× magnification with DIC, ICG only, lysotracker only, and ICG + lysotracker. (B) ROI selected from 40× magnification image and greyscale channels for lysotracker and ICG were used to create a coloured composite view. Colocalization analysis was performed using the Coloc 2 plugin in Fiji ImageJ and showed significant correlation (yellow pixels; Pearson's *r* = 0.8). AU, arbitrary units; ICG, indocyanine green; DIC, differential interference contrast; AF647, Alexa Fluor 647; ROI, region of interest.

Microscopy images of HT‐1080 cells showed significant intracellular uptake of ICG after 30 min and the spatial distribution of ICG was perinuclear (Figure 5A). Significant overlap between the intracellular locations of lysosomes and ICG was observed, as indicated by the yellow pixels (Figure 5B). Colocalization analysis showed a significant correlation between the ICG and lysosome channels with a Pearson's correlation coefficient of 0.8 (Costes *p* value: 1.00).

### Detailed analysis of ICG distribution in patient tumour histology

**Figure 6:**
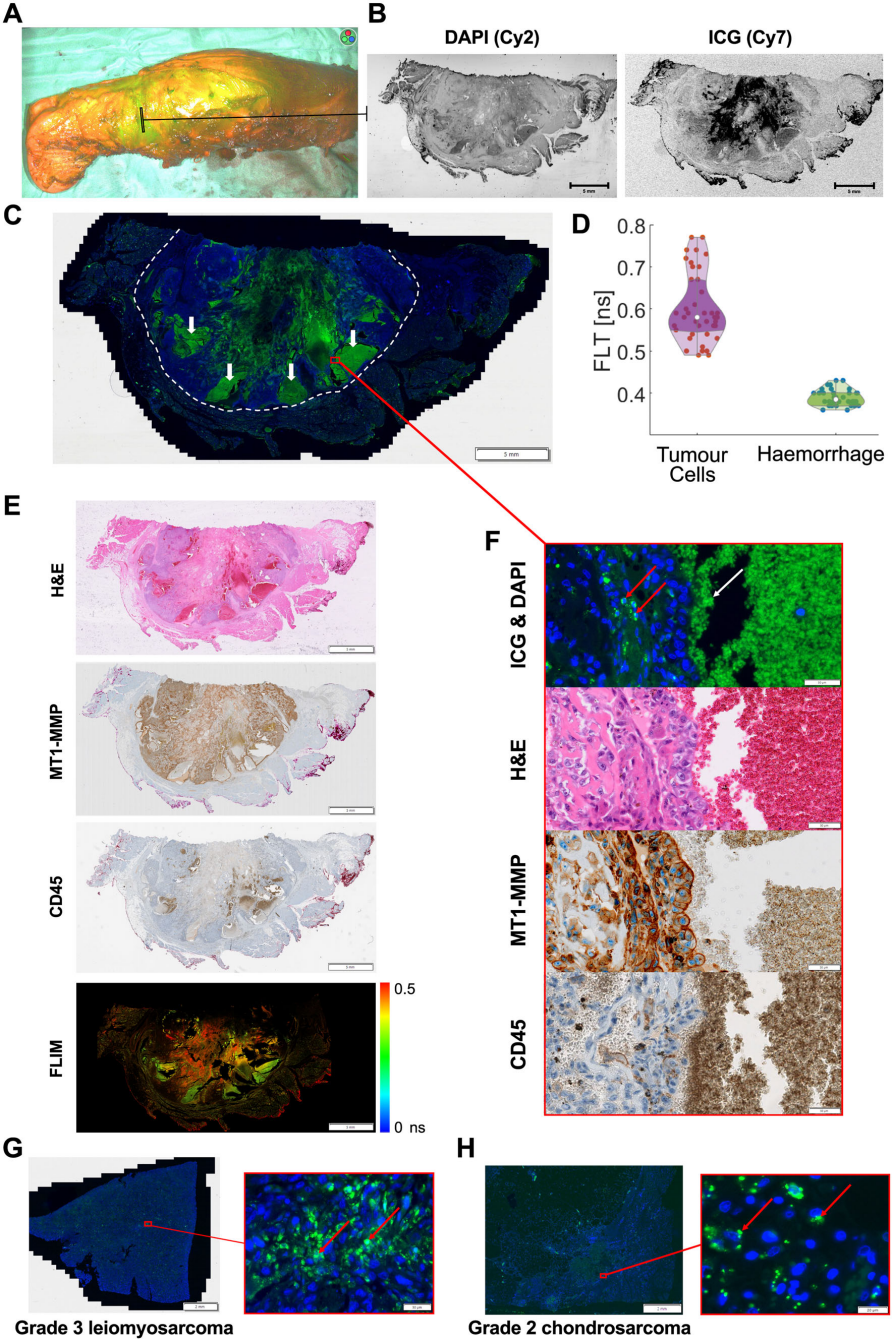
Macroscopic and microscopic analysis of patient tumour sections following FGS with ICG. (A) Macroscopic NIR image of a resected high‐grade osteosarcoma of the femur using the Stryker SPY‐PHI handheld camera, after administration of 75 mg intravenous ICG the day before surgery. A full‐size version of image (A) and a corresponding SPY mode (greyscale) version is provided in supplementary material, Figure S7. (B*) 3‐μm sections processed from the tumour specimen were imaged using the Typhoon Biomolecular Imager; Cy7 channel for ICG, Cy2 channel for DAPI. (C) High‐resolution fluorescence microscopy tissue imaging using Olympus VERSUS200 slide scanner to detect ICG (green) and DAPI stained cell nuclei (blue). The tumour margin is demarcated with a white dotted line, white arrows identify ICG accumulation in areas of haemorrhage and necrosis, red box shows location of ROIs. A full‐size version of image (C) is provided in the supplementary material, Figure S9. (D) Violin plot of FLT values of ICG within tumour cells versus areas of haemorrhage across four representative ROIs (mean value of 0.60 and 0.39 ns, respectively). (E*) 3‐μm sections cut from the same specimen block were stained with H&E, MT1‐MMP antibody (neoplastic sarcoma cell marker), CD45 antibody (immune cell marker) and imaged on a FLIM microscopy system. (F) Higher digital magnification ROIs showing intracellular ICG within tumour cells (red arrows), and ICG accumulation in paucicellular areas (white arrow), alongside the corresponding ROIs with H&E staining, MT1‐MMP IHC and CD45 IHC. (G) High‐resolution fluorescence microscopy tissue imaging of intracellular ICG (green) and DAPI stained cell nuclei (blue) in patient tumour samples from a grade 3 leiomyosarcoma, and a grade 2 chondrosarcoma (H) after administration of 75 mg intravenous ICG (at induction of surgery, and the day before surgery, respectively). The intracellular ICG signal in the leiomyosarcoma and chondrosarcoma was detectable macroscopically (supplementary material, Figure S8). AU, arbitrary units; FLIM, fluorescence lifetime imaging; FLT, fluorescence lifetime; NIR, near‐infrared; ICG, indocyanine; IHC, immunohistochemistry; ROI, region of interest; H&E, haematoxylin and eosin; MT1‐MMP, membrane type‐1 matrix metalloproteinase. *Peripheral signal on (B), (E) is inked margin artefact.

Analysis of a resected high‐grade femoral osteosarcoma following preoperative intravenous administration of 75 mg ICG showed visible fluorescence using the Stryker SPY‐PHI NIR camera (Figure 6A; supplementary material, Figure S7). On analysis of a 3 μm section taken from the tumour margin, biomolecular imaging confirmed the presence of ICG signal (Figure 6B). Subsequent NIR high‐resolution tissue imaging of this section demonstrated intracellular ICG within tumour cells (Figure 6C,F). Histological analysis of two further patient samples, a grade 3 leiomyosarcoma and a grade 2 chondrosarcoma, which both fluoresced intraoperatively during resection (supplementary material, Figure S8), also showed the presence of intracellular ICG (Figure 6G,H). High‐magnification analysis of the osteosarcoma section also showed accumulation of ICG in paucicellular areas of haemorrhage and necrosis, whilst non‐tumour tissue had a pattern of low non‐specific extracellular punctate signal (Figure 6C; supplementary material, Figure S9). The area of high ICG accumulation on fluorescence microscopy corresponded to tumour tissue on H&E staining (Figure 6E). IHC with a sarcoma cell marker (MT1‐MMP), a membrane‐bound proteinase overexpressed in sarcoma tissue and cell lines, showed that regions of *in vivo* ICG cellular uptake corresponded to neoplastic cells (Figure 6F). Further IHC with a CD45 immune cell marker showed that the neoplastic cells with intracellular retention of ICG are negative for CD45. These results strongly suggest that ICG cellular positivity to be neoplastic, rather than in cells of immune cell origin. To further demonstrate that ICG is present in two distinct locations within the tumour (malignant cells versus areas of haemorrhage), FLIM quantification was performed across four ROIs within the osteosarcoma section. The mean fluorescence lifetime (FLT) values for ICG within tumour cells was longer than for ICG within areas of haemorrhage; 0.60 ns versus 0.39 ns, respectively (Figure 6D). The FLT values obtained are typical of ICG within tumour and non‐tumour conditions, as previously shown [45].

## Discussion

Tumour uptake of ICG is described as non‐selective, since the dye does not exhibit specific cancer‐cell targeting moiety [39], unlike targeted dyes; however, some studies suggest that ICG does possess inherent affinity to cancer cells *via* its ability to bind to cell membranes [42, 53]. Although several mechanisms have been suggested to explain the preferential tumour uptake of ICG, including tight junction disruption, the expression of specific membrane transporters and dysregulated endocytic pathways, the exact mechanisms are still not fully understood [21, 42]. It is appreciated that the *in vivo* pharmacokinetics of ICG uptake in tumours will differ greatly from *in vitro* studies, as factors such as biodistribution, serum protein binding, tumour vascularity, the EPR effect, and patient heterogeneity will influence the uptake and availability of ICG to tumour cells. Furthermore, factors such as neoadjuvant radiotherapy may influence the distribution and retention of ICG within treated tumours and surrounding normal tissue. The timing of administration of ICG preoperatively will also have a large effect on tumour retention and will influence the signal‐to‐background ratio at the time of resection [31].

Previously, the EPR effect has been proposed to play a key role in tumour accumulation of ICG compared with healthy tissue. However, some studies have suggested that passive tumour cell targeting of ICG and preferential ICG uptake and retention by individual tumour cells also have a significant role [42]. Our results build on this prior work, with a focus on sarcoma, and suggest that the rate of ICG uptake differs depending on the cancer cell type, with more proliferative cell lines having the highest uptake within 30 min. Our *in vitro* data showed that ICG cellular uptake increased with greater incubation time up to 24 h, with no further signal increase seen at 48 h. We propose that this is due to lysosomal saturation of ICG occurring around the 24 h timepoint, when maximum cellular uptake is achieved. This supports the current clinical use and proposed administration regime of injecting ICG 12–24 h before surgery to optimise tumour fluorescence intraoperatively [6, 29]. Taken together, these *in vitro* studies support the concept of passive tumour cell targeting of ICG *via* increased cellular uptake and retention, due to dysregulated endocytosis in high‐grade cancer cells [42].

We observed a significant correlation between ICG cellular uptake and the cell line proliferation rate. The proliferation capacity of a cancer cell line is often related to marked genetic alterations, dysregulated cellular pathways, and the presence of activated oncogenes, contributing to enhanced metabolic processes and high invasive potential [52]. The increased uptake of ICG observed in more proliferative cell lines can be postulated to be due to dysregulated clathrin‐mediated endocytosis (CME) and endosomal trafficking. Aberrant endocytic pathways in tumour cells are a recognised hallmark of cancer [54], leading to enhanced internalisation and dysregulation between the lysosomal pathway and the recycling route [55].

Our data suggest that CME is an important mechanism of ICG cellular uptake in a dedifferentiated chondrosarcoma cell line. In this study, a cell‐permeable CME inhibitor significantly reduced the uptake of ICG in the highly proliferative HT‐1080 sarcoma cell line. However, at higher ICG concentrations, the level of PS2 inhibition was reduced. This suggests that other endocytic mechanisms such as caveolae‐dependent endocytosis may also play a role [56], but at lower concentrations of ICG, cells are largely dependent on CME for effective ICG uptake. Further supporting this, PS2 did not completely inhibit ICG cellular uptake, even with a high concentration of the inhibitor and a low concentration of ICG. Recent studies have demonstrated that PS2 also inhibits clathrin‐independent endocytosis, suggesting only partial selectivity of this compound for CME [56, 57]. Organic‐anion‐transporting polypeptide (OATP) transporters also have a role in ICG cellular uptake, with OATP1B3 reported as a major ICG transporter in liver cells [58]. However, OATP1B3 is not widely expressed in sarcoma tissue and other members of the OATP family have not been shown to have a role in ICG uptake [59]. On fluorescence microscopy analysis, the spatial distribution of ICG was perinuclear and ICG significantly colocalized with lysosomes within 30 min. The entrapment of ICG within the membrane trafficking system is likely to account for its longer retention time within tumour cells compared with normal tissue [42], and the lysosomal environment may also result in higher stability and a reduced degradation rate of ICG. The effects of the lysosomal environment on the retention of ICG and its spectral properties is an area that warrants further research.

Following the surgical resection of a high‐grade osteosarcoma, a grade 3 leiomyosarcoma and a grade 2 chondrosarcoma, ICG was detectable within the tumour macroscopically and microscopically on 3 μm tumour sections. High‐resolution NIR imaging identified intracellular ICG within sarcoma cells across three patient tumour sections (with varying timepoints of ICG administration), supporting active tumour cell uptake of ICG. The neoplastic nature of the cells retaining ICG was demonstrated using IHC with a sarcoma marker (MT1‐MMP) and immune cell marker (CD45). These histological findings are in keeping with results from our *in vitro* uptake studies. In the osteosarcoma sample, the predominant accumulation of ICG was observed within areas of haemorrhage and necrosis, which supports the EPR effect as an important mechanism for ICG retention within tumours. This is supported by a previous study of synovial sarcoma by Sardar *et al*, who compared the fluorescence distribution of ICG to an EGFR targeted agent and showed high ICG signal intensity in acellular tumour areas [44]. Quantitative analysis using FLIM microscopy further demonstrated that the spatial distribution of ICG within the tumour was present in two distinct areas: intracellularly within tumour cells with long FLTs, and in extracellular areas of haemorrhage with shorter FLTs. FLIM is an advancing technology based on a photophysical quantity, which measures the time ICG spends in its excited state. Since FLIM is largely unaffected by ICG concentration [60], it allowed us to interrogate the locality of ICG based on the local tissue environment, despite a greater amount of the dye being confined to the acellular areas, as visualised on normal fluorescence imaging. Our clinical data demonstrate that FLT imaging can delineate tumours from normal tissue despite non‐specific ICG accumulation, thereby enhancing the translational potential of ICG FLT imaging for sarcomas [45]. Further studies are underway to understand the mechanisms behind the enhanced FLT in sarcomas and its connection with the cellular uptake mechanisms discussed above. Its utility for FGS in oncological surgery is currently an active area of investigation [45, 60].

The relative amount of ICG that accumulates within acellular areas compared to cellular uptake will differ greatly between tumours, depending on the degree of haemorrhage and necrosis. Although further work is required to fully understand the complex cellular mechanisms of ICG uptake in tumour cells, there is likely to be significant variability depending on the cancer type, locality, grade, size, and vascularity. For the clinical use of ICG in sarcoma resection, the fluorescence of ICG should be used only as a visual guide and should not mandate resection. The decision to resect tissue based on fluorescence should, therefore, remain at the surgeon's discretion and operative experience. Although the scope of this work is focused on sarcoma, it is likely that the mechanisms described will also apply to other solid cancer types in the context of fluorescence‐guided surgery with ICG. We also hypothesise that FGS may play a future role in the histological margin assessment of sarcomas based on *in vivo* ICG cellular uptake.

A limitation of this study involves the analysis of only three patient tumour samples, due to the labour‐intensive processing and high‐resolution NIR microscopy of FGS tissue samples. However, it provided important data to support the *in vitro* work by demonstrating the spatial orientation of ICG in three different sarcoma subtypes. Future work with larger patient cohorts will enable robust statistical analysis to further explore variability in ICG uptake across sarcoma subtypes.

In conclusion, this study found that cellular ICG uptake correlated significantly with the cell line proliferation rate *in vitro*, with maximum ICG uptake observed after 24 h. Our data support CME as an important pathway for ICG cellular uptake in sarcoma, resulting in ICG being trafficked into lysosomes. High‐resolution NIR tissue imaging and FLIM quantification in human tumour specimens was able to differentiate the presence of ICG within sarcoma cells from ICG in paucicellular areas of haemorrhage. Taken together, we propose that the increased uptake and accumulation of ICG in sarcomas is driven by a synergistic mechanism involving the EPR effect combined with active tumour cell endocytosis, with maximum cellular uptake occurring at the 24 h timepoint. We theorise that both mechanisms will be enhanced in intermediate‐to‐high‐grade tumours due to their genetic aberrations, increased proliferation rate, and a highly disorganised tumour environment [61]. By improving our understanding of the mechanisms of ICG uptake in sarcoma, this study aims to maximise the translational benefit of this technology for clinical use.

## Author contributions statement

CDC and KSR conceived the study. CDC, MJB, TAP, RT and KSR were involved with data acquisition. CDC, MJB, TAP, RT, KSR, RP, ATNK and JCK performed data analysis, image analysis, and image quantification. CDC and TAP created the final figures. KSR and ADB were involved with data interpretation and provided expert clinical information on patient histology analysis. CDC, MJB and KSR wrote the initial article. All authors reviewed, edited, and contributed significantly to the article. All authors approved the final version of the article.

## Prior submissions

An article containing some early results of this work was published on the preprint server bioRxiv (https://doi.org/10.1101/2021.04.05.438013) in April 2021, to support the clinical investigational use of ICG at our centre. This updated article contains new significant findings that were not previously published, or under consideration for publication elsewhere.

## Supporting information


Supplementary materials and methods
**Figure S1**. Image Quantification using Zen 3.3 Blue Edition Software (Zeiss)
**Figure S2**. FLIM microscopy with four representative ROIs selected for quantification of ICG mean fluorescence lifetime (FLT) and intensity, in areas of haemorrhage versus tumour cells
**Figure S3**. Example of how FACS data was processed for each sample
**Figure S4**. ICG cellular uptake FACS data generated across all cell lines
**Figure S5**. Flow cytometry data for PS2 treatment on ICG uptake at 25 μm and 10 μm
**Figure S6**. Supplementary microscopy images demonstrating the colocalization of ICG (green) and Lysotracker (red) in HT‐1080 cells at 20× and 40× magnification, with digital magnification of cell ROIs
**Figure S7**. Widefield NIR images of a high‐grade osteosarcoma of the femur
**Figure S8**. Widefield NIR images of resected sarcoma specimens taken intraoperatively demonstrating macroscopic tumour fluorescence
**Figure S9**. Enlarged image of high‐resolution fluorescence microscopy tissue imaging shown in Figure 6C
**Table S1**. Information on the cell lines used in this study
**Table S2**. The seeding densities of each cell line to achieve consistent 80%–90% confluency across all cell lines at point of experimentation (after 48 h)
**Table S3**. Primary antibody and reagent details for IHC staining of patient histology slides

## Data Availability

The data that support the findings of this study are available from the corresponding author upon reasonable request.
