# Supplementary material for: Investigating the mechanisms of indocyanine green tumour uptake in sarcoma cell lines and ex vivo human tissue
Source: J Pathol. 2025 Sep 10;267(3):315–28. doi: 10.1002/path.6473 (PMC12531117; doi:10.1002/path.6473)
Supplement: Supplementary file 1 — Supplementary materials and methods Figure S1. Image Quantification using Zen 3.3 Blue Edition Software (Zeiss) Figure S2. FLIM microscopy with four representative ROIs selected for quantification of ICG mean fluorescence lifetime (FLT) and intensity, in areas of haemorrhage versus tumour cells Figure S3. Example of how FACS data was processed for each sample Figure S4. ICG cellular uptake FACS data generated across all cell lines Figure S5. Flow cytometry data for PS2 treatment on ICG uptake at 25 μm and 10 μm Figure S6. Supplementary microscopy images demonstrating the colocalization of ICG (green) and Lysotracker (red) in HT‐1080 cells at 20× and 40× magnification, with digital magnification of cell ROIs Figure S7. Widefield NIR images of a high‐grade osteosarcoma of the femur Figure S8. Widefield NIR images of resected sarcoma specimens taken intraoperatively demonstrating macroscopic tumour fluorescence Figure S9. Enlarged image of high‐resolution fluorescence microscopy tissue imaging shown in Figure 6C Table S1. Information on the cell lines used in this study Table S2. The seeding densities of each cell line to achieve consistent 80%–90% confluency across all cell lines at point of experimentation (after 48 h) Table S3. Primary antibody and reagent details for IHC staining of patient histology slides [file PATH-267-315-s001.docx]

**Investigating the mechanisms of indocyanine green tumour uptake in sarcoma cell lines and *ex vivo* human tissue**

CD Chan *et al. J Pathol* <https://doi.org/10.1002/path.6473>

**Supplementary materials and methods**

**Supplementary Figures S1–S9**

**Supplementary Tables S1–S3**

**Supplementary materials and methods**

**Cell culture**

All cell lines used in this study are adherent but differ in their morphology and proliferation rate. Cells were passaged when they reached 80–90 % confluency in T-75 cell culture flasks (Corning Life Sciences, Flintshire, UK), using 1.5 ml of prewarmed 1× trypsin 0.5% Trypsin-EDTA 10× (Gibco, London, UK). Trypsinised cells were neutralised in 10 ml of fresh media and centrifuged at 150× g for 5 min. The supernatant was aspirated, and the cell pellet reconstituted in 10 ml fresh media and added to a new flask.

**Cell preparation in chamber slides**

Cells were seeded at a density between 2 × 10^4^ and 4 × 10^4^ cells/well in Nunc^®^ Lab-Tek^®^ II CC2 chamber slides (Thermo Fisher Scientific, Leicestershire, UK) and left to establish for 48 h at 37 °C. The initial seeding densities were calculated based on each cell lines’ proliferation rate (supplementary material, Table S2) to ensure a comparable number of cells at the time of experimentation. After 48 h, the media was aspirated and cells were incubated in ICG in media (400 µl) for either 15 or 30 min. ICG-containing media was removed, and cells washed with media ×2, before being fixed with 400 µl 4% paraformaldehyde (PFA) solution (pH 6.9, buffered, Sigma-Aldrich, Dorset, UK) for 10 min at room temperature. PFA was removed, cells washed ×3 in PBS, the chamber system was removed as per the manufacturer’s instructions, and the cells were stained with DAPI Aqueous Fluoroshield mounting medium (Abcam, Cambridge, UK) before adding a glass coverslip. All slides were imaged immediately after mounting.

**Flow cytometry**

Flow cytometric analysis of cellular ICG fluorescence was performed on the BD FACSCanto™ II Cell Analyser, using the 635 nm laser with bandwidth 780/60. Samples were analysed at a low flow rate for 50,000–100,000 total events. Flow cytometry data were analysed using FlowJo v10.8 Software (Becton, Dickinson and Company, Franklin Lakes, NJ, USA). The main cell population was gated on SSC-A/FSC-A, doublets discriminated using FSC-H/FSC-A, and a histogram for 635;780/60-A generated with log scaling. The median fluorescence intensity (MFI) for each cell line was calculated by (MFI = MFI ICG Positive Sample – MFI Unstained Sample). MFI values were obtained using the statistical function within FlowJo v10.8 software. The experiment was repeated on three separate occasions.

**Fluorescence microscopy**

Microscope System: Zeiss Axioimager 1 (Carl Zeiss Microscopy Ltd, Cambridge, UK). Camera: Zeiss MRm (Monochrome). Light Source: HXP120 metal halide, 825 µW +/− 1.65 µW. ICG detection: Semrock Cy7-B cube bandpass filter: excitation 670–745 nm, emission 768–850 nm (exposure time 2,000–15,000 ms, light source intensity 100%). Lysotracker Deep Red (Invitrogen, Paisley, UK) detection: Alexa Fluor 647 channel, Zeiss 50 cube; bandpass filter: excitation 625–655 nm, emission 665–715 nm (exposure time 200 ms, light source intensity 100%). DAPI detection: Zeiss 49 Cube bandpass filters: excitation 335–385 nm, emission 420–470 nm (exposure time 80 ms, light source intensity 100%). Differential interference contrast (DIC) light source: TL VIS-LED Lamp (light source intensity 10%), exposure time 400 ms. Zeiss Lenses: 20× Plan-Apochromat 20×/0.8 M27; 40× EC Plan-Neofluar 40×/0.75 M27. All fluorescent images taken with NIR mode active and 1×1 pixel binning. All samples were imaged immediately after preparation.

**Colocalisation analysis**

The ICG and lysosome channels were analysed in Fiji ImageJ (v.2.3.0/1.53, https://imagej.net/software/fiji/downloads, date last accessed 05/01/2025) using the Coloc 2 plugin. The paired images (40× magnification) were first median filtered (radius = 2) followed by a background subtraction with a rolling ball radius of 50 pixels. The resulting images were employed in a colocalization analysis using the Coloc 2 plugin with individual image thresholds automatically defined by Costes regression. Additionally, a point spread function (PSF) of 3 pixels and Costes randomization of 10 pixels were used in the analysis. The resulting 2D intensity histogram of the ICG and lysosome channels with a regression line showed the colocalization coefficient. The Pearson’s R value was 0.8 and Spearman’s rank correlation value was 0.81.

**High-resolution tissue imaging and biomolecular imaging**

Patient ICG specimen sections were imaged using an Olympus VS200 slide scanner (Olympus, Hamburg, Germany) equipped with an X-Cite Xylis light engine, AHF pentaband beam splitter, ORCA-Flash4.0 V3 monochrome camera, and the following single-band excitation and emission filters for DAPI; 352–404 nm/416–452 nm, and ICG; 721–749/767–849 nm. Sections with H&E were imaged using a 20× 0.8NA XAPO objective and system-defined autoexposure. ICG-containing sections were imaged using a 40× 1.4NA XAPO objective and 4 ms and 1 s exposure time for DAPI and ICG, respectively. Image histograms were adjusted, and images subsequently processed and exported using Olympus OlyVIA software v3.4.1 (Olympus). CD45 IHC specimen slides were imaged at 20× using a Leica Aperio CS2 slide scanner (Leica Biosystems, Vista, CA, USA).

**Patient specimen slides**

Three tissue sections were cut from FFPE tissue blocks at 3 µm thickness using a Leica RM2245 microtome and mounted on SuperFrost Plus™ (Thermo Fisher Scientific) adhesive slides. Slides for MT1-MMP IHC and DAPI counterstaining were baked at 60 °C for 60 min prior to staining. H&E staining was performed on a Dako CoverStainer automated staining platform (Agilent Technologies, Cheshire, UK). For IHC staining, the primary MT1-MMP antibody (MAB3328/MMP-14 clone LEM-2/15.8, Merck Life Science UK Limited, Dorset, UK) was diluted 1:2,500 to the working concentration using Da Vinci Green universal diluent (Biocare Medical, Pacheco, CA, USA). The CD45 antibody (M0701/ clone 2B11 + PD7/26, Agilent Technologies) was diluted 1:250 to the working concentration. All IHC staining was performed on the Ventana^®^ Discovery Ultra automated IHC staining platform (Roche Diagnostics, West Sussex, UK). For antigen retrieval, Discovery CC1 (pH 8.5, Roche Diagnostics) was applied at 100 °C for 32 min. The primary MT1-MMP antibody incubation time was 32 min, and the secondary antibody incubation time was 16 min. The primary CD45 antibody incubation time was 32 min, and the secondary antibody incubation time was 12 min. Chromomap DAB was used as the chromogen, with haematoxylin nuclear counterstain. Following washing, slides were glass-mounted using Pertex^®^ mounting medium (Atom Scientific, Manchester, UK). For detection of ICG, DAPI counterstain-only slides were prepared and stained on the Ventana^®^ Discovery Ultra platform (Roche Diagnostics). No antigen retrieval step was applied, only dewaxing. Three drops of Discovery QD DAPI counterstain (Roche Diagnostics) were applied to each slide and incubated for 40 min. Following washing, slides were glass-mounted using ProLong™ fluorescent mounting medium (Thermo Fisher). Reagent details are provided in the supplementary material, Table S3.


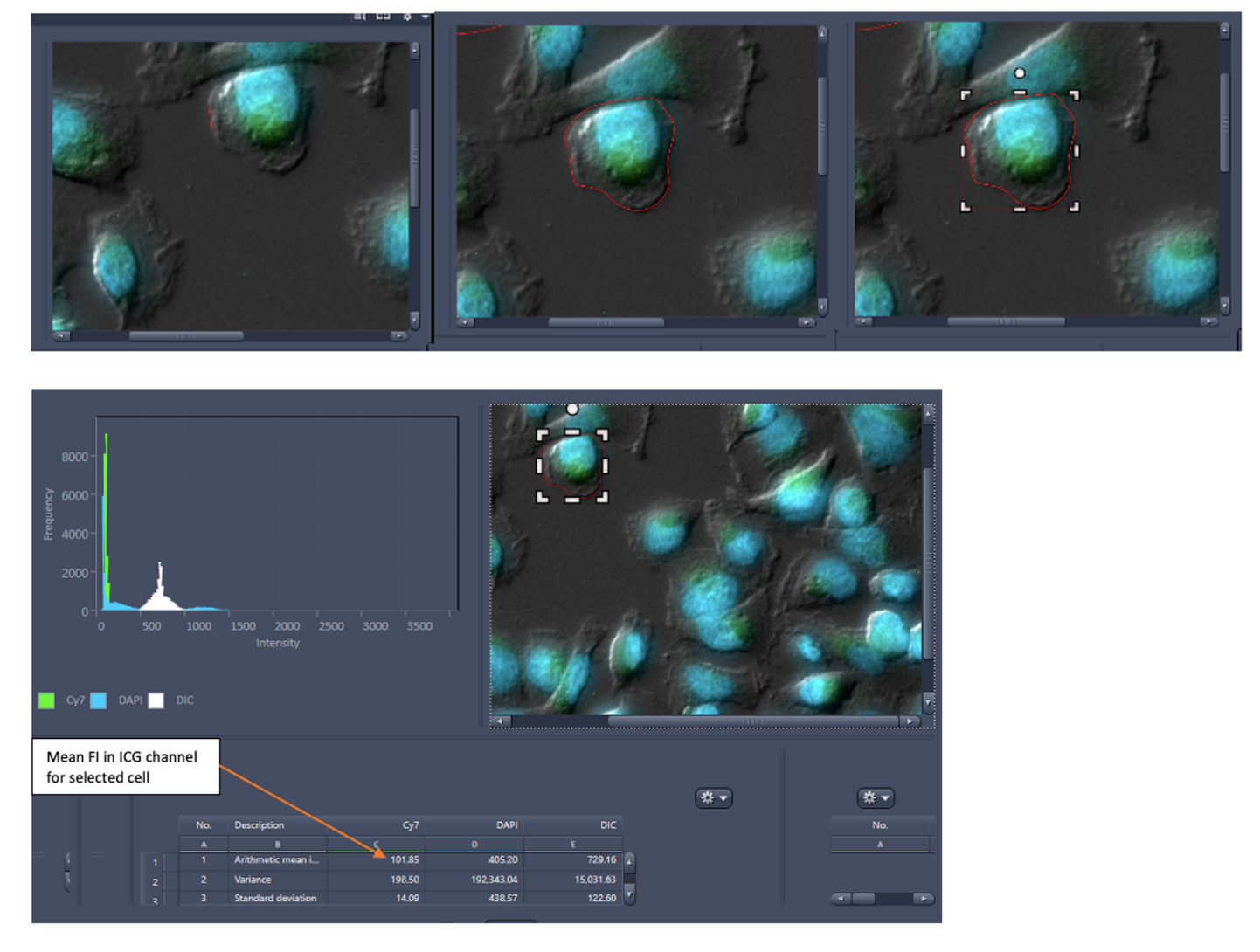


**Figure S1. Image quantification using Zen 3.3 Blue Edition Software (Zeiss).** The Zen software polygonal tool was used to draw around each cell at 40× magnification using DIC to guide the identification of individual cells. The mean florescence intensity (MFI) per cell was generated within the Cy7 column of the table on the histogram image view tab. Four representative cells were analysed per FOV with this method, and the average MFI calculated. Screenshots were taken from ZEN 3.3 Blue Edition software (Zeiss Group Headquarters, Oberkochen, Baden-Württemberg, Germany).


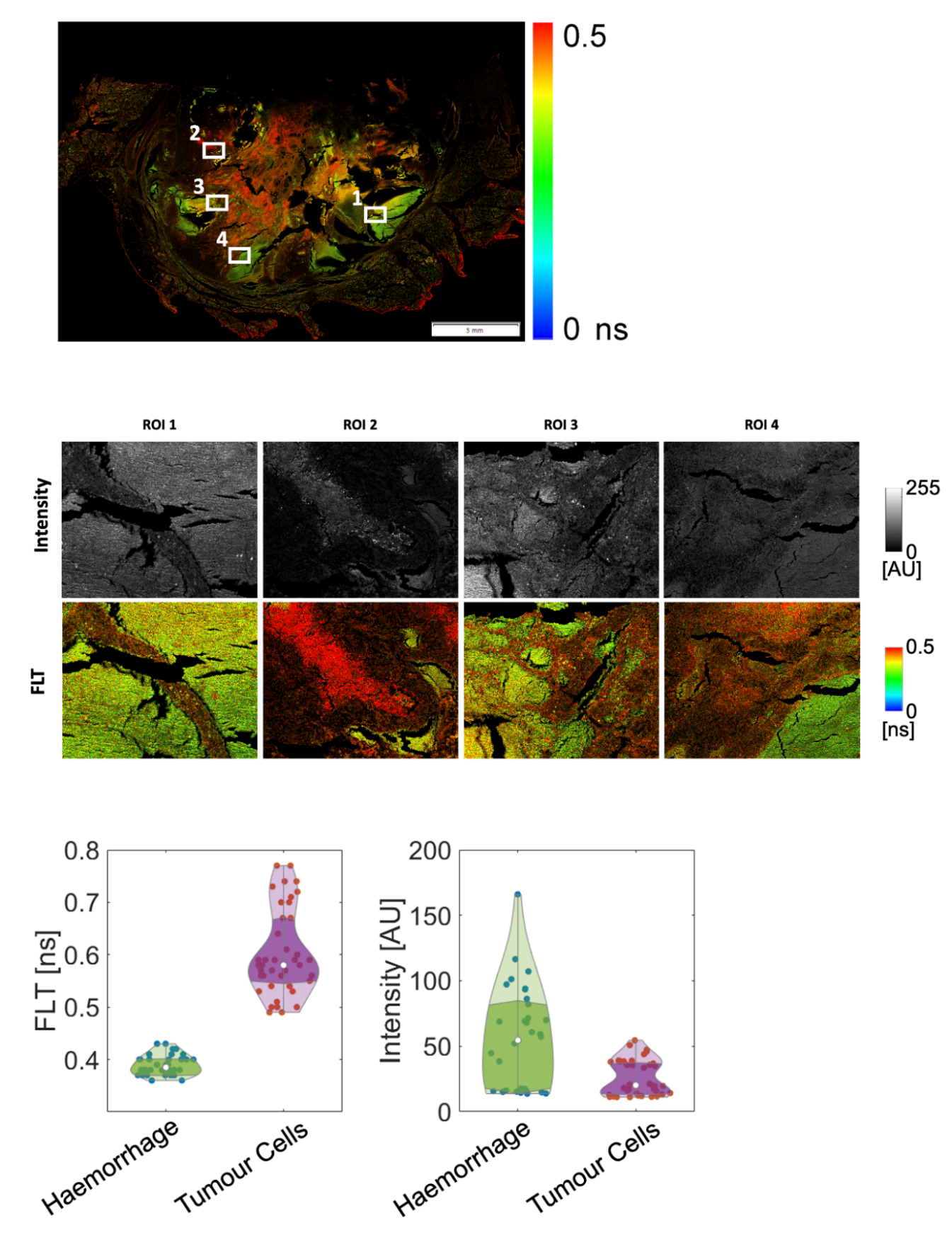


**Figure S2.** **FLIM microscopy with four representative ROIs selected for quantification of ICG mean fluorescence lifetime (FLT) and intensity, in areas of haemorrhage versus tumour cells.** Additional comparison of FLT to intensity is shown in this supplemental figure, with the FLT violin plot taken from Figure 6 in the main article. A total of 74 smaller ROIs were selected across the four large ROIs to produce the violin plots for both FLT and fluorescence intensity. Colour bars represent intensity value (AU) and lifetime value in nanoseconds for intensity and FLT imaging, respectively.


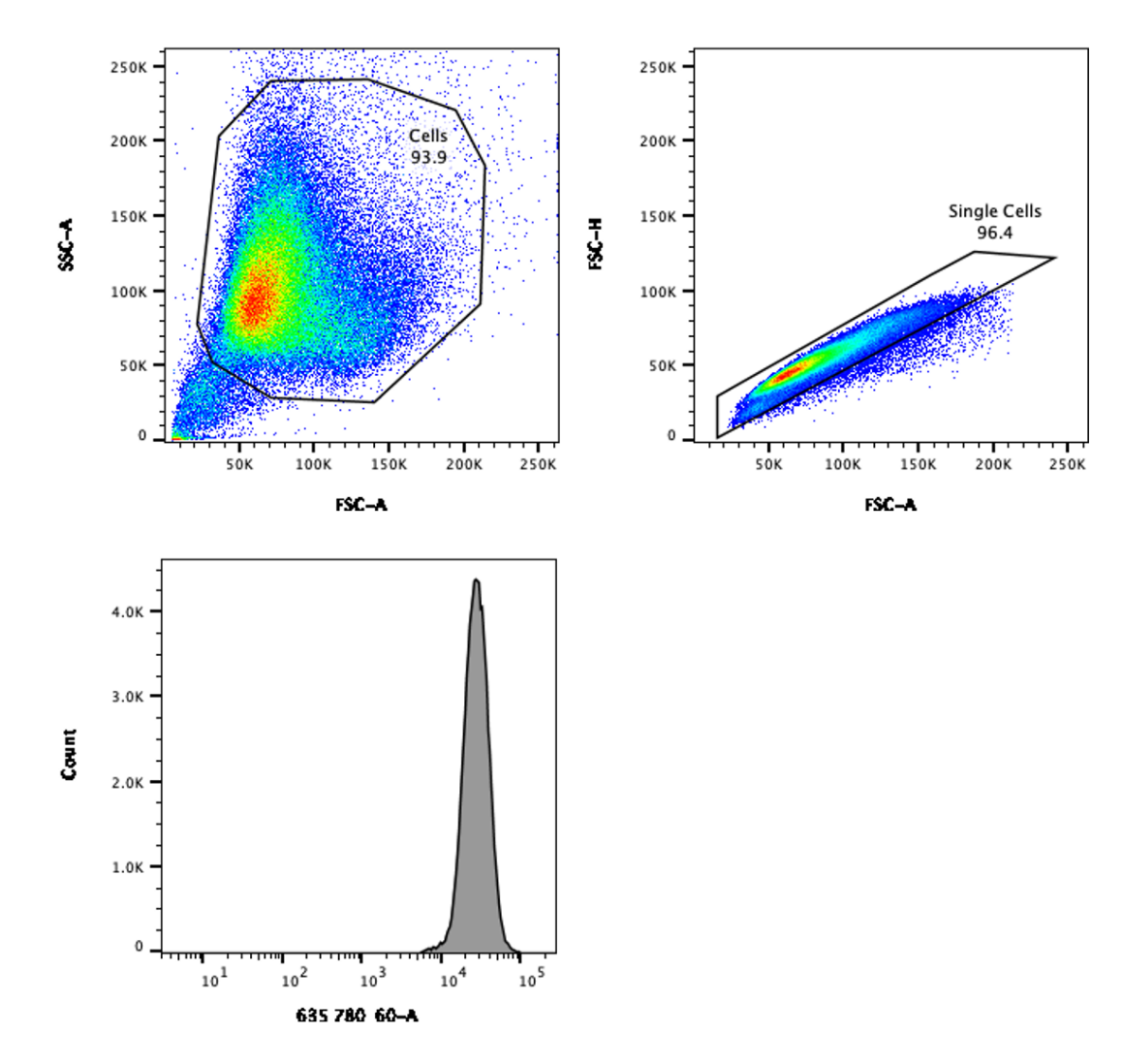


**Figure S3.** **Example of how FACS data was processed for each sample.** The main cell population was gated on SSC-A/FSC-A, doublets were discriminated via gating on FSC-H/FSC-A, and intensity of 635; 780/60 nm displayed as a histogram (*x*-axis log scale). All analyses were performed using FlowJo software (v10.8).


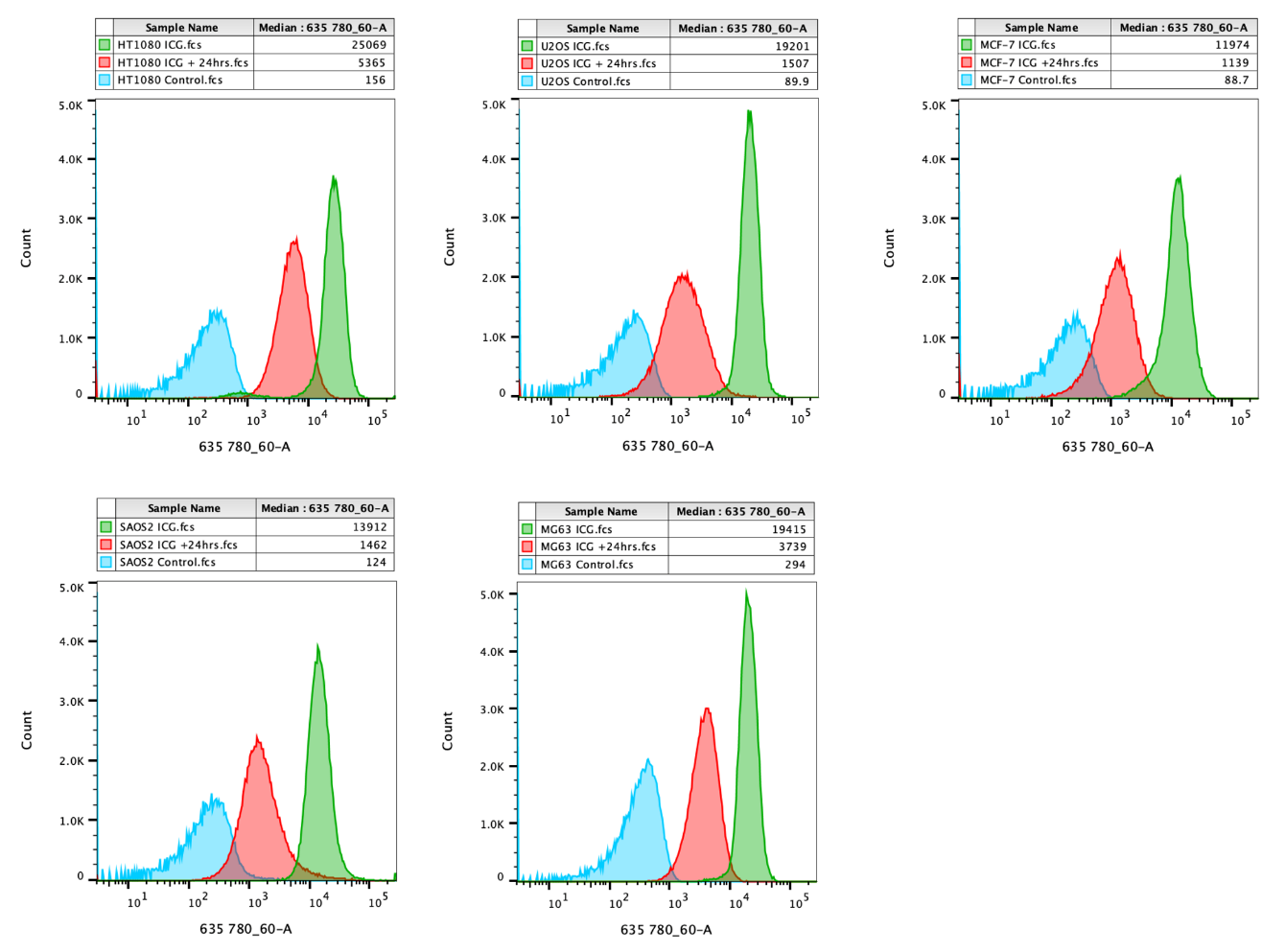


**Figure S4.** **ICG cellular uptake** **FACS data generated across all cell lines.** Cells were incubated in 25 µM ICG for 30 min and then analysed by flow cytometry (green). For the 24 h retention experiment ICG was removed after 30 min and then cells were incubated for an additional 24 h in fresh media prior to analysis (red). Data were analysed using FlowJo v10.8 Software for MacOS (Becton, Dickinson and Company, Franklin Lakes, NJ, USA).


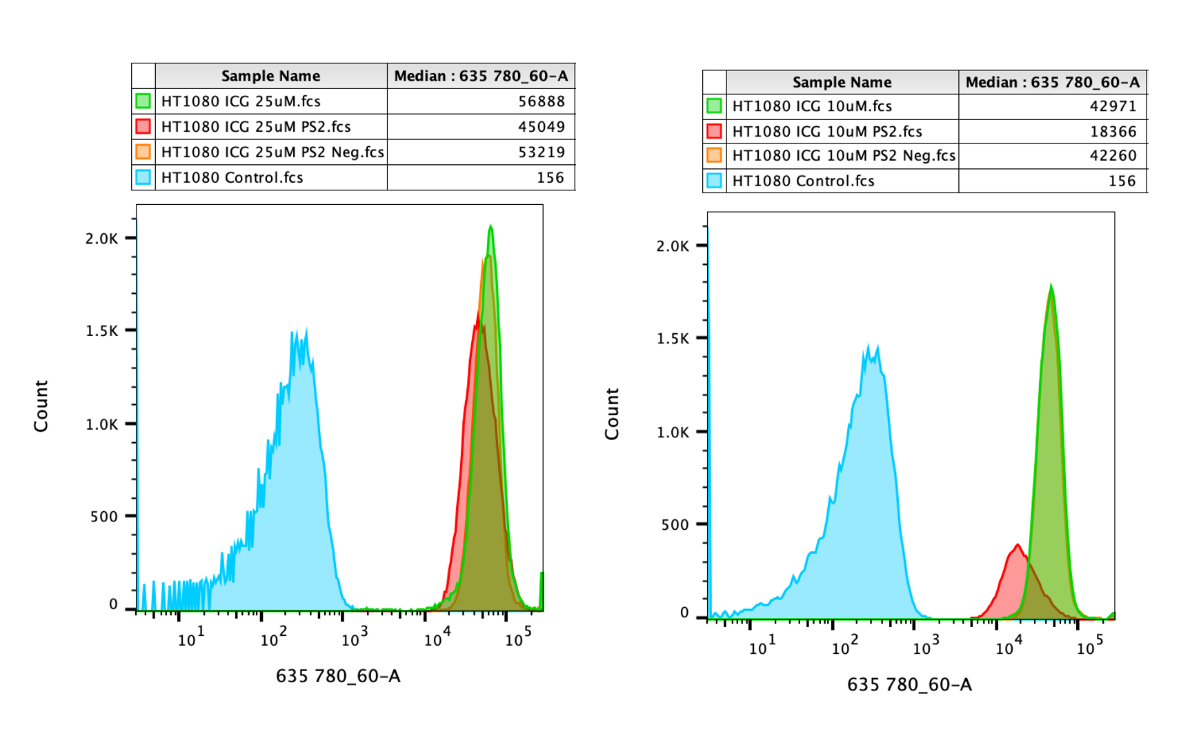


**Figure S5.** **Flow cytometry data for PS2 treatment on ICG uptake at 25 µM and 10 µM.** Experiments performed on the HT-1080 cell line, pretreated with 30 µM PS2 or 30 µM PS2 negative control for 15 min, followed by incubation with ICG for 30 min in the continued presence of the inhibitor. Data were analysed using FlowJo v10.8 Software for MacOS.


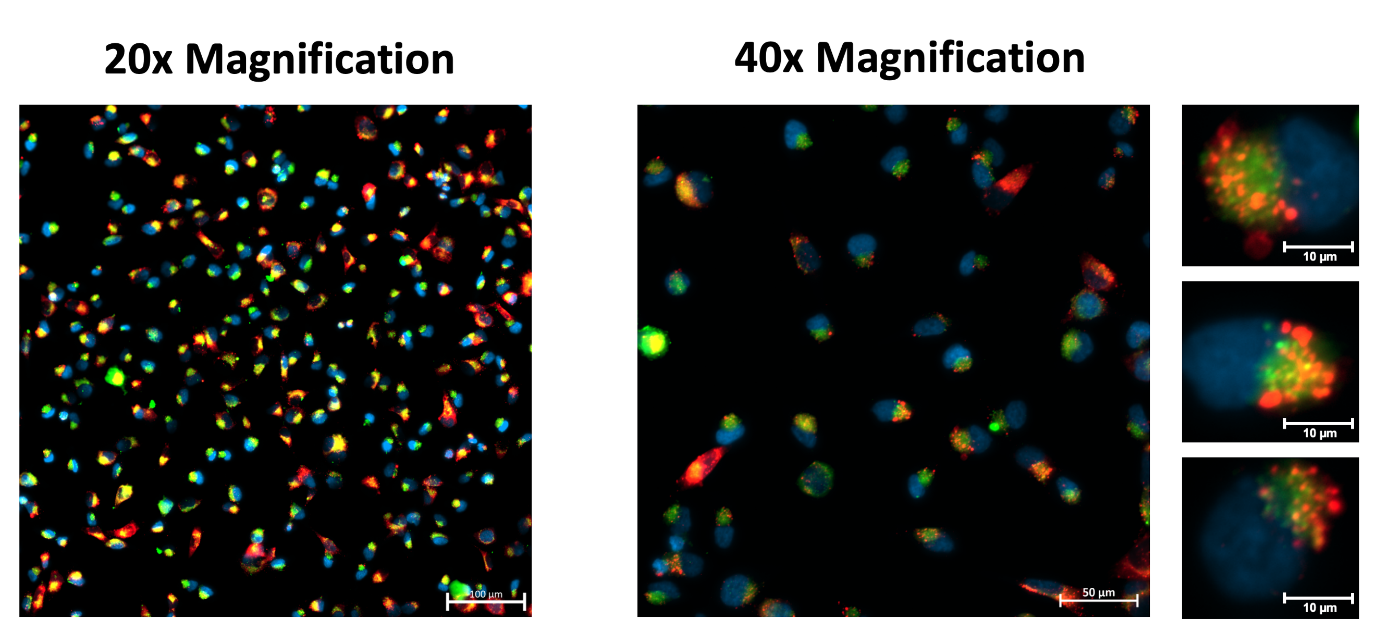


**Figure S6.** **Supplementary microscopy images demonstrating the colocalization of ICG (green) and Lysotracker (red) in HT-1080 cells at 20× and 40× magnification, with digital magnification of cell ROIs.** Cells were incubated in chamber slides with 50 nm lysotracker for 1 h followed by 10 µM ICG for 30 min. Cells were fixed with 4% PFA and stained with a DAPI nuclear stain (blue).


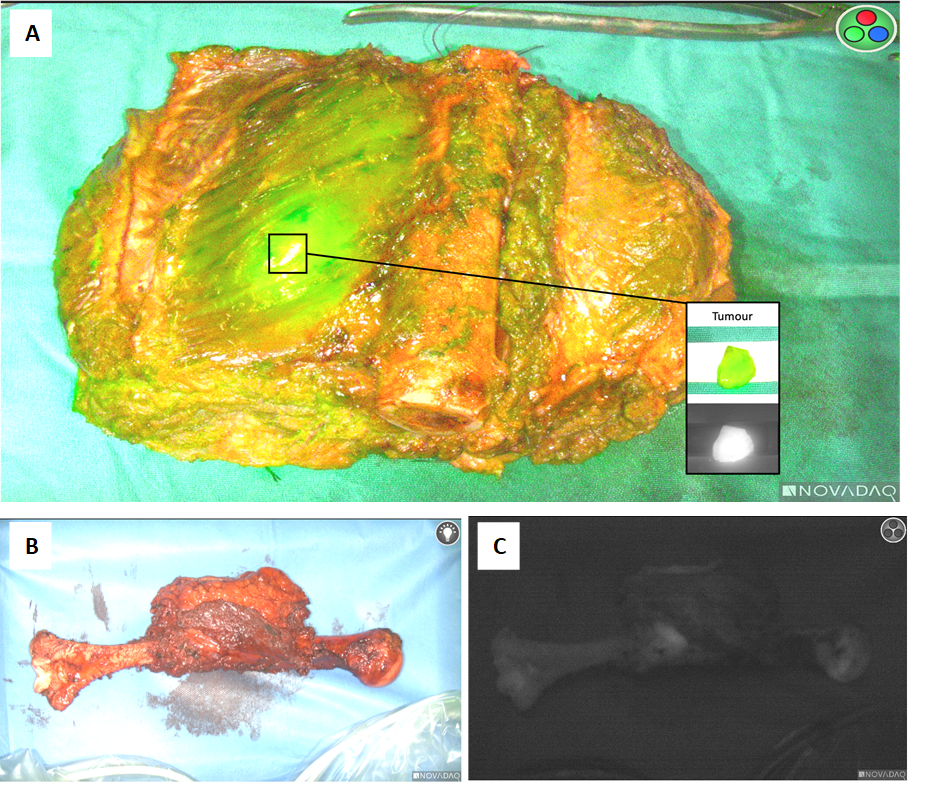


**Figure S7.** **Widefield NIR images of a high-grade osteosarcoma of the femur.** (A) Macroscopic NIR fluorescence overlay image of a resected high-grade osteosarcoma of the femur using the Stryker SPY-PHI handheld camera, after administration of 75 mg intravenous ICG the day before surgery, as used in Figure 6A. Further supplementary image (B) of the same specimen, taken using NIR SPY mode (greyscale) with the Stryker SPY-PHI camera. Image (A) from the main article has been reused here to allow for direct comparison with the SPY mode supplementary image (B).


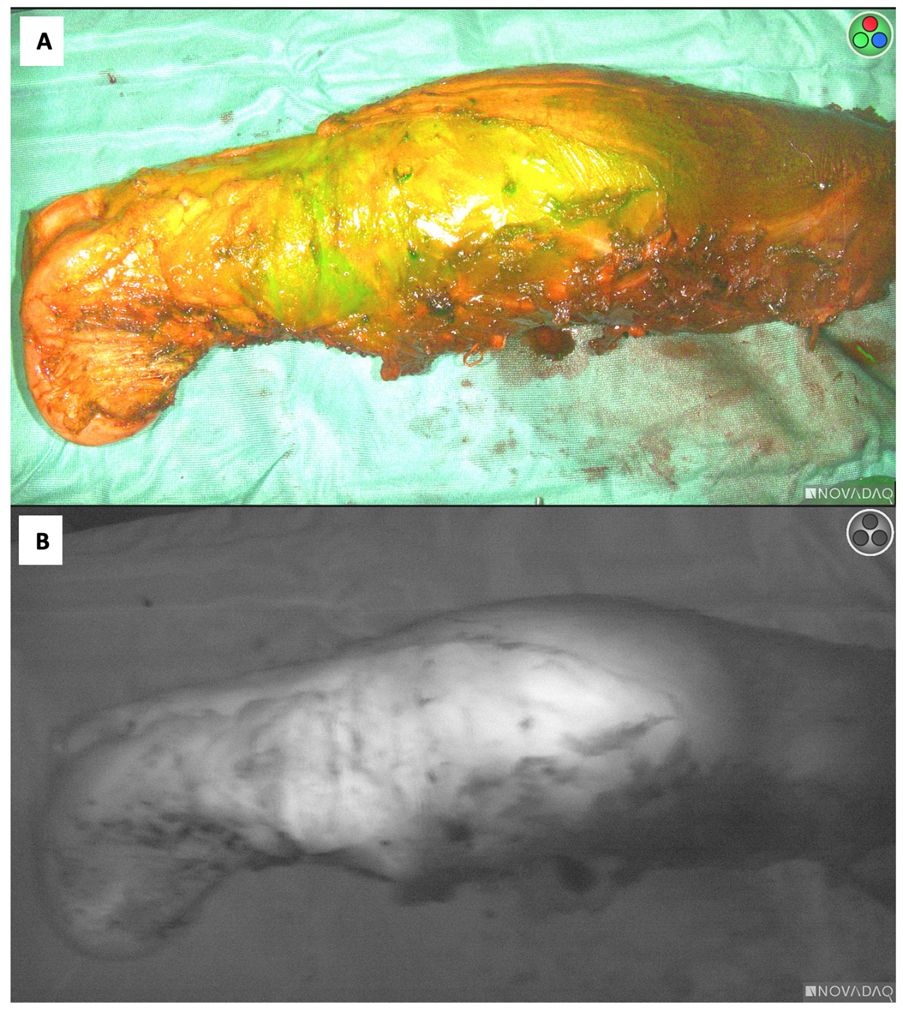


**Figure S8.** **Widefield NIR images of resected sarcoma specimens taken intraoperatively demonstrating macroscopic tumour fluorescence. (**A) Widefield image of the grade 3 leiomyosarcoma taken using the Stryker SPY-PHI NIR camera (Stryker Corp, Kalamazoo, MI, USA) intraoperatively on the specimen table, showing macroscopic fluorescence of the tumour component (green) in fluorescence overlay mode. The tissue block was then imaged in both overlay mode and SPY mode, prior to sections been cut for histological analysis in Figure 6G. Macroscopic brightfield image of (B) the resected grade 2 chondrosarcoma of the humerus, (C) alongside a NIR SPY mode image showing tumour fluorescence, taken using the Stryker SPY-PHI camera.

**
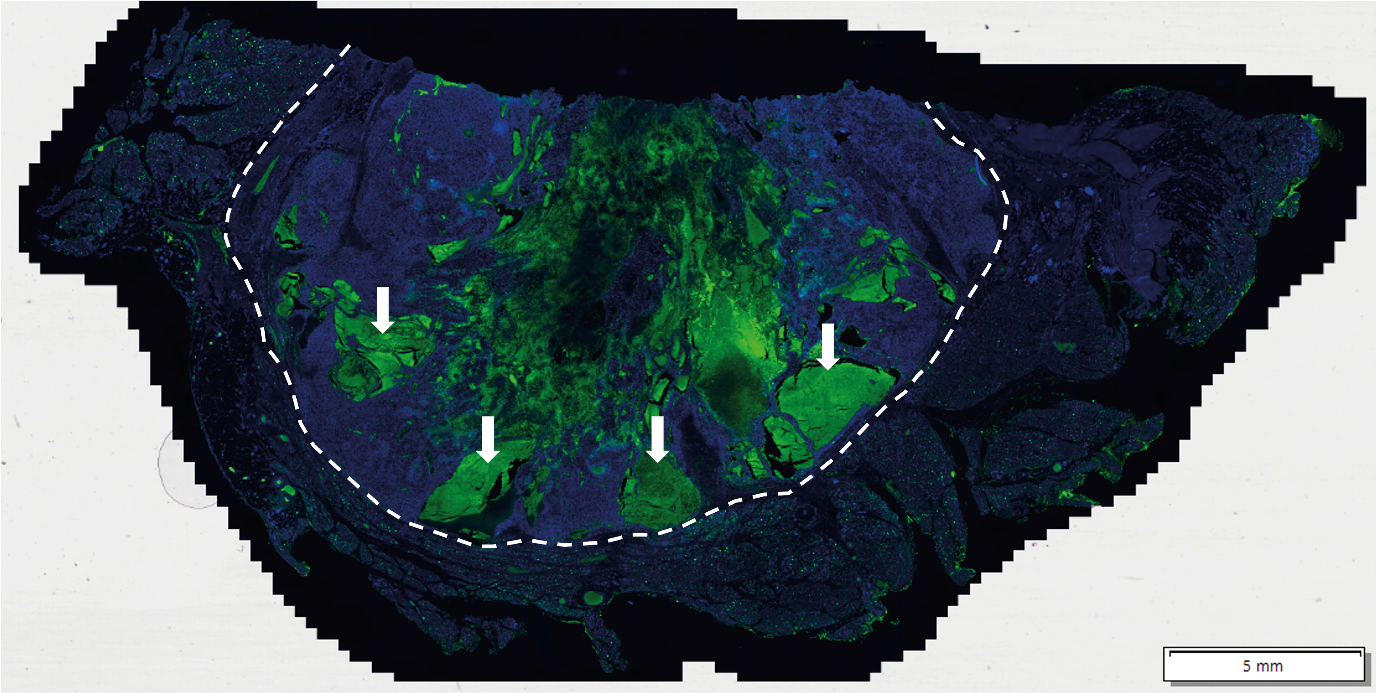
**

**Figure S9.** **Enlarged image of high-resolution fluorescence microscopy tissue imaging shown in Figure 6C.** The tumour margin is demarcated with a white dotted line, white arrows identify ICG accumulation in areas of haemorrhage and necrosis. The image was acquired using an Olympus VS200 slide scanner to detect ICG (green) and DAPI stained cell nuclei (blue).

**Table S1.** Information on the cell lines used in this study

| **Cell Line** | **Origin** | **Doubling time (h)** |
| --- | --- | --- |
| HT-1080 | ATCC (Manassas, VA, USA) | 26 |
| U2OS | ATCC | 29 |
| SaOS-2 | ATCC | 43 |
| MG-63 | Sheffield University, (Sheffield, UK) | 28 |
| MCF-7 | ATCC | 35–40 |

ATCC, American Type Culture Collection.

**Table S2.** The seeding densities of each cell line to achieve consistent 80–90% confluency across all cell lines at point of experimentation (after 48 h)

| **Cell Line** | **Seeding Density cells/chamber**  (Nunc^®^ Lab-Tek^®^ II CC2 chamber slides) | **Seeding Density cells/well**  (Corning^®^ 6-well plate) |
| --- | --- | --- |
| HT-1080 | 20,000 | 250,000 |
| U2OS | 24,000 | 300,000 |
| SaOS-2 | 37,000 | 462,500 |
| MG-63 | 27,000 | 337,500 |
| MCF-7 | 37,000 | 462,500 |

**Table S3.** Primary antibody and reagent details for IHC staining of patient histology slides

| **Antibody** | **Clone** | **Species** | **Manufacturer** | **Product Reference** | **Working Dilution** |
| --- | --- | --- | --- | --- | --- |
| MT1-MMP (Anti-MMP-14) | LEM-2/15.8 | Mouse | Merck Life Science UK Limited, Dorset, UK | MAB3328 | 1:2,500 |
| CD45, Leucocyte Common Antigen (Autostainer Link 48) | 2B11 + PD7/26 | Mouse | Agilent Technologies, Cheshire, UK. | M0701 | 1:250 |

| **Reagent** | **Name** | **Supplier** | **Product ref.** |
| --- | --- | --- | --- |
| HRP Inhibitor | Discovery Inhibitor | Roche Diagnostics, West Sussex, UK | 760-4840 |
| Secondary antibody | Ventana^®^ ultraView HRP Multimer | Roche Diagnostics | 253-4290 |
| Antibody diluent | Da Vinci Green Diluent | Biocare Medical (California, USA) | PD900L |
| Staining buffer | Reaction Buffer | Roche Diagnostics | 950-300 |
| Deparaffinisation Buffer | Discovery Wash | Roche Diagnostics | 950-510 |
| Antigen Retrieval solution | Discovery CC1 | Roche Diagnostics | 950-500 |
| Liquid Coverslip | Ultra LCS | Roche Diagnostics | 650-210 |
| Chromogen | Discovery ChromoMap DAB | Roche Diagnostics | 760-159 |
| Counterstain | Discovery QD DAPI | Roche Diagnostics | 760-4196 |
| Counterstain | Haematoxylin II | Roche Diagnostics | 790-2208 |
| Counterstain | Bluing Reagent | Roche Diagnostics | 760-2037 |
